# Novel Common Variants Associated with Obesity and Type 2 Diabetes Detected Using a cFDR Method

**DOI:** 10.1038/s41598-017-16722-6

**Published:** 2017-11-27

**Authors:** Qiang Zhang, Ke-Hao Wu, Jing-Yang He, Yong Zeng, Jonathan Greenbaum, Xin Xia, Hui-Min Liu, Wan-Qiang Lv, Xu Lin, Wei-Dong Zhang, Yuan-Lin Xi, Xue-Zhong Shi, Chang-Qing Sun, Hong-Wen Deng

**Affiliations:** 1 College of Public Health, Zhengzhou University, Zhengzhou, NO.100 Kexue Road, High-Tech Development Zone Of States, Zhengzhou, P.R. China; 2 Center for Bioinformatics and Genomics, School of Public Health and Tropical Medicine, Tulane University, New Orleans, LA 70112 USA; 3 Department of Endocrinology and Metabolism, the Third Affiliated Hospital of Southern Medical University, Guang Zhou, P.R. China; 4 College of Sciences, Beijing Jiao Tong University, Beijing, China

## Abstract

Genome-wide association studies (GWASs) have been performed extensively in diverse populations to identify single nucleotide polymorphisms (SNPs) associated with complex diseases or traits. However, to date, the SNPs identified fail to explain a large proportion of the variance of the traits/diseases. GWASs on type 2 diabetes (T2D) and obesity are generally focused on individual traits independently, and genetic intercommunity (common genetic contributions or the product of over correlated phenotypic world) between them are largely unknown, despite extensive data showing that these two phenotypes share both genetic and environmental risk factors. Here, we applied a recently developed genetic pleiotropic conditional false discovery rate (cFDR) approach to discover novel loci associated with BMI and T2D by incorporating the summary statistics from existing GWASs of these two traits. Conditional Q-Q and fold enrichment plots were used to visually demonstrate the strength of pleiotropic enrichment. Adopting a cFDR nominal significance level of 0.05, 287 loci were identified for BMI and 75 loci for T2D, 23 of which for both traits. By incorporating related traits into a conditional analysis framework, we observed significant pleiotropic enrichment between obesity and T2D. These findings may provide novel insights into the etiology of obesity and T2D, individually and jointly.

## Introduction

Genome-wide association studies (GWASs) have successfully identified hundreds of SNPs associated with complex diseases or traits. However, to date, the SNPs identified fail to explain a large proportion of the variance of the traits/diseases under study. Previous studies have suggested that GWASs have the potential to explain a larger proportion of “missing heritability”^1,2^ mainly by using larger sample sizes^3^. However, although acquiring larger sample sizes may increase statistical power, it is often not feasible since the recruiting and genotyping of additional participants is too costly. Therefore, there is a need for analytical methods that can better and more efficiently utilize the information contained in the existing pool of available data for the identification of trait-associated loci. Several of these types of methods have recently been developed^4–6^ and successfully applied^7,8^ to identify novel loci for various complex traits.

Pleiotropy is the phenomenon of a single gene or locus affecting two or more phenotypes^9^. There is ample evidence to suggest that genetic pleiotropy exists in many correlated diseases and traits, such as bipolar disorder and schizophrenia^10^, indicating that related traits may share overlapping genetic mechanisms. Through the incorporation of information regarding genetic pleiotropy, we can improve the detection power of common variants associated with complex diseases or traits by effectively increasing the sample sizes without the need to recruit more individuals. The joint analysis of related phenotypes may reveal novel insights into the common biological mechanisms and overlapping pathophysiological relationships between complex traits.

Andreassen *et al*.^4^ developed a genetic-pleiotropy-informed conditional false discovery rate (cFDR) method by leveraging two GWASs from associated traits in a conditional analysis. The method has been successfully applied to genetically associated diseases and phenotypes including schizophrenia and bipolar disorder^7^, as well as blood pressure and other phenotypes^8^. Our group has recently successfully applied the cFDR method to the joint analyses of bone mineral density (BMD) and breast cancer^11^, BMD and coronary artery disease^12^, femoral neck (FNK) BMD and height^13^, and T2D and birth weight^14^. All of these studies improved statistical power through the joint analysis of related traits, and unambiguously demonstrated the utility of the method for improving the identification of potentially novel trait-associated variants.

Obesity is a chronic metabolic disorder mainly characterized by excessive body fat. Body Mass Index (BMI) is widely used in obesity research and clinical diagnosis to quantify an individual’s tissue mass. Identification of the genetic determinants for BMI, a non-invasive measure of obesity that predicts the risk of related complications^15^, could lead to a better understanding of the biological basis of obesity. Epidemiological studies estimate that the prevalence of overweight/obese individuals increased by >40% between 1980 and 2013^16^, and that these elevated obesity levels are a driving force for the similarly rapid increase of Type II Diabetes (T2D)^17^. T2D is a chronic metabolic disorder characterized by high blood sugar, insulin resistance, and relative lack of insulin, all of which share some genetic susceptibility and functional mechanisms with obesity^18^. Heritability studies have demonstrated a substantial genetic contribution to both obesity risk (h^2^~40–70%)^19^ and T2D (h^2^~26–69%)^20^. In 2014, an estimated 387 million people were living with diabetes, corresponding to a worldwide prevalence of 8.3%, and 90% of these individuals had T2D^21^.

There has been substantial evidence to indicate an important relationship between obesity and T2D, along with strong support^22,23^ to suggest that obesity and T2D share some common genetic risk factors. The accumulation of body fat may be associated with several conditions related to T2D including insulin resistance, hyperinsulinemia, the reduced utilization of glucose in muscles and other tissues, and impaired glucose tolerance^24^. Additionally, Corbin *et al*.^25^ used Mendelian Randomization (MR) Egger analysis to explore the complex relationship between these traits and demonstrated a true causal effect of BMI on T2D, as well as potential pleiotropy between the two phenotypes. Although dozens of genetic loci associated with BMI or T2D have been detected by GWASs^26,27^, these loci can explain at best 10% of the genetic variance for either obesity^28^ or T2D^29^. Considering the high degree of heritability and potential pleiotropy between these phenotypes, the two traits are ideal for the further analyses using the cFDR approach to improve the detection of loci associated with obesity and/or T2D.

In this study, we applied the genetic-pleiotropy-informed cFDR method^4^ on two large datasets of GWAS summary statistics for BMI and T2D^30,31^ to identify novel loci and pleiotropic relationships between these traits. These two GWASs have identified 97 and 62 loci associated with BMI and T2D respectively, but they only explain 2.7% and 5.7% of the total heritability for these traits^30,31^. The purpose of our study is to improve SNP detection for obesity and T2D using these two existing GWASs, and gain some novel insights into the shared biological mechanisms and overlapping genetic heritability between them. The clarification of potentially shared genetic determinants may have significant implications for the identification of important biomarkers and development of novel therapeutic approaches for joint prediction, prevention, and intervention of the two related diseases/phenotypes.

## Results

### Assessment of pleiotropic enrichment

**Figure 1:**
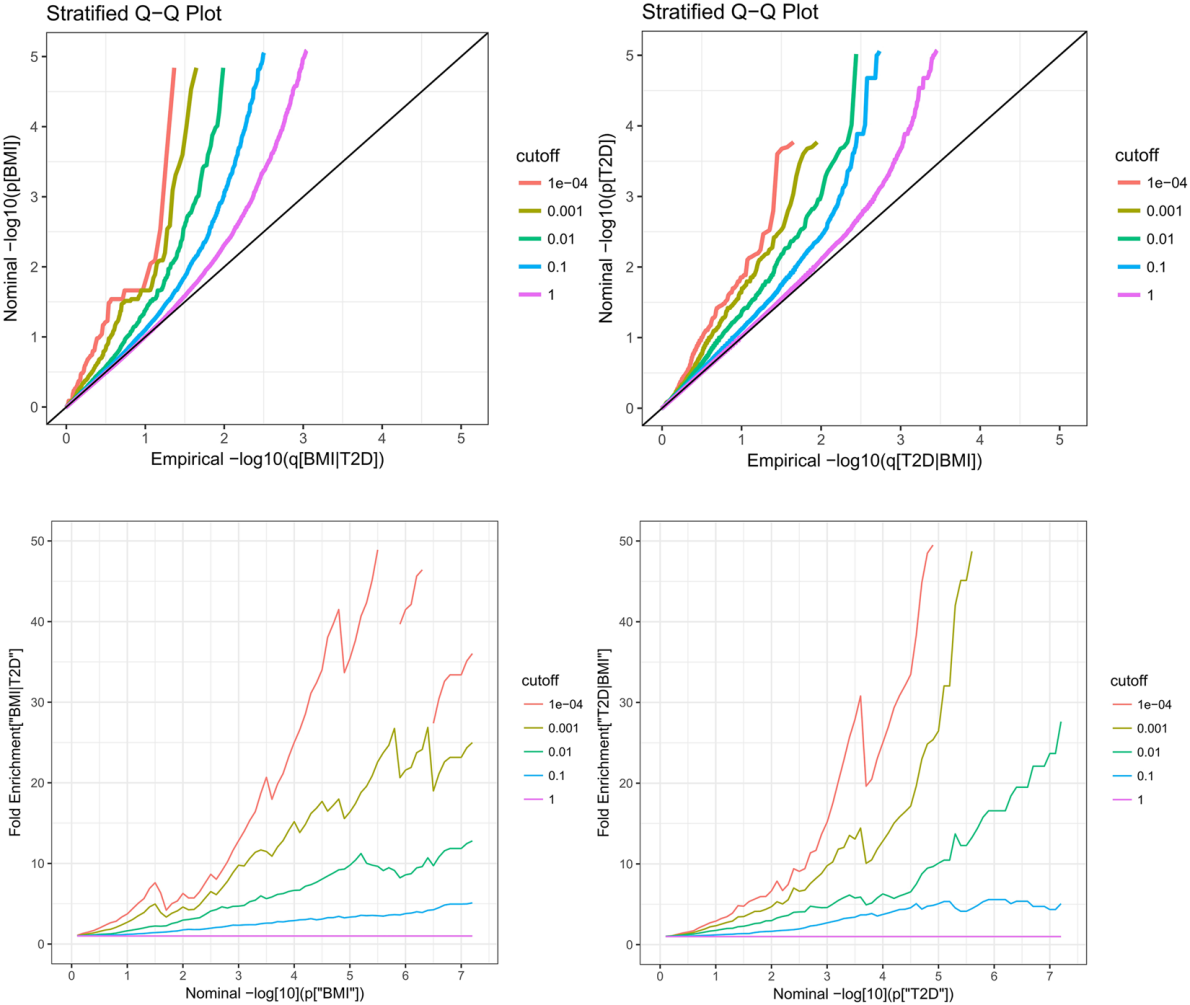
Stratified QQ (upper panel) and Enrichment (lower panel) plots. Upper Panel: Stratified QQ plots of nominal versus empirical –log_10_ p-values in (left) BMI as a function of significance of the association with T2D, and in (right) T2D as a function of significance of the association with BMI. Lower Panel: Fold-enrichment plots of enrichment versus nominal –log10 p-values for (left) BMI below the standard GWAS threshold of p < 5 × 10^−8^ as a function of significance of the association with T2D, and (right) T2D below the standard GWAS threshold of p < 5 × 10^−8^ as a function of significance of the association with BMI. The purple line with slope of zero represents all SNPs.

The conditional Q-Q plot for BMI conditional on T2D (Upper Panel (left) in Fig. 1) showed some enrichment across varying significance thresholds for T2D. The presence of leftward shift when restricting the analysis to include the SNPs that have more significant associations with BMI indicates an increase in the number of true associations for a given T2D p-value. Similar enrichment is observed for T2D given BMI (Upper Panel (right) in Fig. 1), as there appears to be a similar departure pattern between the different curves. These leftward deflections from the null line indicate a greater proportion of true associations for any given BMI nominal p-value.

Based on the fold-enrichment plot (Lower Panel of Fig. 1), we observed SNP enrichment for BMI across different levels of significance with T2D and *vice versa*. For progressively stringent p-value thresholds for BMI SNPs, we observed about a 50-fold increase in the proportion of SNPs reaching the genome wide significance level of -log10 (p) > 7.3 when comparing the subset with the most stringent conditional association to the group with all SNPs. A 50-fold increase was also observed for T2D.

As negative controls, conditional Q-Q plots for BMI given nominal p-values of association with attention-deficit/hyperactivity disorder (ADHD) (Upper Panel (left) in Figure S1) and major depressive disorder (MDD) (Lower Panel (left) in Figure S1), and T2D conditional on ADHD (Upper Panel (left) in Figure S2) and MDD (Lower Panel (left) in Figure S2) all showed no enrichment and *vice versa*.

### BMI loci identified with cFDR

**Figure 2:**
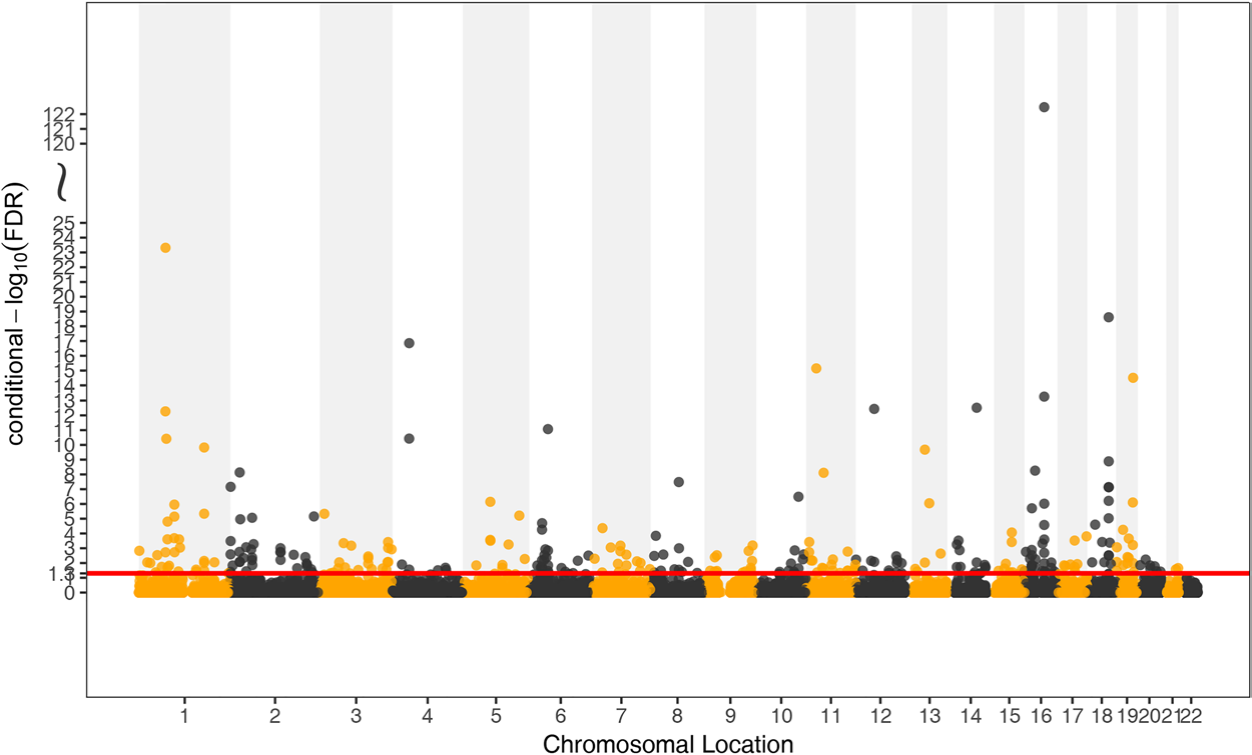
Conditional Manhattan plot of conditional –log_10_ FDR values for BMI given T2D (BMI|T2D). The red line marks the conditional –log_10_ FDR value of 1.3 corresponds to a cFDR < 0.05.

Conditional on their association with T2D, we identified 287 significant SNPs (cFDR < 0.05) for BMI variation (Fig. 2 and Table S2), which were mapped to 21 different chromosomes (1–21) and annotated to 323 genes. In the original meta-analysis for BMI GWAS^31^, 105 SNPs had p-values smaller than 1 × 10^−5^ while 36 of them reached the standard genome-wide significance of 5 × 10^−8^. We confirmed 43 SNPs that were reported in the original BMI GWAS analysis^31^ and previous BMI related GWASs^32^. Another 40 SNPs that were reported to be associated with BMI-related traits were also confirmed in our analysis^32–34^. The rest of the 204 SNPs were not previously reported in the original BMI GWAS^31^ or any other previous obesity studies. However, 26 of these 204 SNPs are in high linkage disequilibrium (LD) (r2 > 0.6) with other BMI-associated SNPs reported previously (Table S3). Among the 323 genes these 287 SNPs were annotated to, 146 of these genes were newly detected compared to the original BMI GWAS^31^ and previous obesity-related studies (Table S2). Among all the 287 detected loci for BMI, most of the genes were enriched in BMI-related terms such as “positive regulation of cellular metabolic process”, “positive regulation of metabolic process” and “regulation of protein metabolic process”. GO term enrichment analysis results were detailed in Table 1.

**Table 1:** Functional Term Enrichment Analysis.

Pathway ID	Pathway description	Count in gene set	False discovery rate
**BMI GO:0031325**	positive regulation of cellular metabolic process	58	0.00983
**GO:0031328**	positive regulation of cellular biosynthetic process	41	0.0119
**GO:0007275**	multicellular organismal development	74	0.0172
**GO:0045935**	positive regulation of nucleobase-containing compound metabolic process	39	0.019
**GO:0051173**	positive regulation of nitrogen compound metabolic process	40	0.0197
**GO:0010604**	positive regulation of macromolecule metabolic process	53	0.0223
**GO:0010628**	positive regulation of gene expression	38	0.0223
**GO:0051130**	positive regulation of cellular component organization	30	0.0223
**GO:0051254**	positive regulation of RNA metabolic process	35	0.0223
**GO:0045859**	regulation of protein kinase activity	21	0.0282
**GO:0009893**	positive regulation of metabolic process	63	0.0306
**GO:0010557**	positive regulation of macromolecule biosynthetic process	36	0.0316
**GO:0051246**	regulation of protein metabolic process	48	0.0343
**GO:0051338**	regulation of transferase activity	24	0.0379
**GO:0044767**	single-organism developmental process	77	0.0449
**GO:0035270**	endocrine system development	8	0.0462
**T2D GO:0031016**	pancreas development	6	0.00523
**GO:0009749**	response to glucose	6	0.0222
**GO:0035270**	endocrine system development	6	0.0222
**GO:0061017**	hepatoblast differentiation	2	0.0289
**GO:0031018**	endocrine pancreas development	4	0.0393
**GO:0000976**	transcription regulatory region sequence-specific DNA binding	10	0.0116
**GO:0044212**	transcription regulatory region DNA binding	11	0.0116
**GO:0043565**	sequence-specific DNA binding	12	0.0214
**GO:0051427**	hormone receptor binding	5	0.0478
**BMI and T2D GO:0010883**	regulation of lipid storage	3	0.0231
**GO:0070344**	regulation of fat cell proliferation	2	0.007
**GO:0007267**	cell-cell signaling	15	8.79e–6
**GO:0016055**	Wnt signaling pathway	10	2.38e–6
**GO:0198738**	cell-cell signaling by wnt	10	2.38e–6
**GO:1905114**	cell surface receptor signaling pathway involved in cell-cell signaling	10	3.41e–6
**GO:0045444**	fat cell differentiation	5	0.00279
**GO:0045598**	regulation of fat cell differentiation	5	0.000541
**GO:0015908**	fatty acid transport	4	0.000926
**GO:0015909**	long-chain fatty acid transport	4	0.000246
**GO:0019395**	fatty acid oxidation	4	0.00268
**GO:0030258**	lipid modification	4	0.0485
**GO:0034440**	lipid oxidation	4	0.00277
**GO:0030308**	negative regulation of cell growth	7	3.11e–5
**GO:0045600**	positive regulation of fat cell differentiation	4	0.000457
**GO:0010565**	regulation of cellular ketone metabolic process	4	0.0239
**GO:0019217**	regulation of fatty acid metabolic process	4	0.00394
**GO:0045834**	positive regulation of lipid metabolic process	4	0.0146
**GO:0045923**	positive regulation of fatty acid metabolic process	4	0.000242
**GO:0046320**	regulation of fatty acid oxidation	4	0.000145
**GO:0046321**	positive regulation of fatty acid oxidation	4	2.78e–5
**GO:0050872**	white fat cell differentiation	4	4.02e–5
**GO:0050873**	brown fat cell differentiation	4	0.000653

### T2D gene loci identified with cFDR

**Figure 3:**
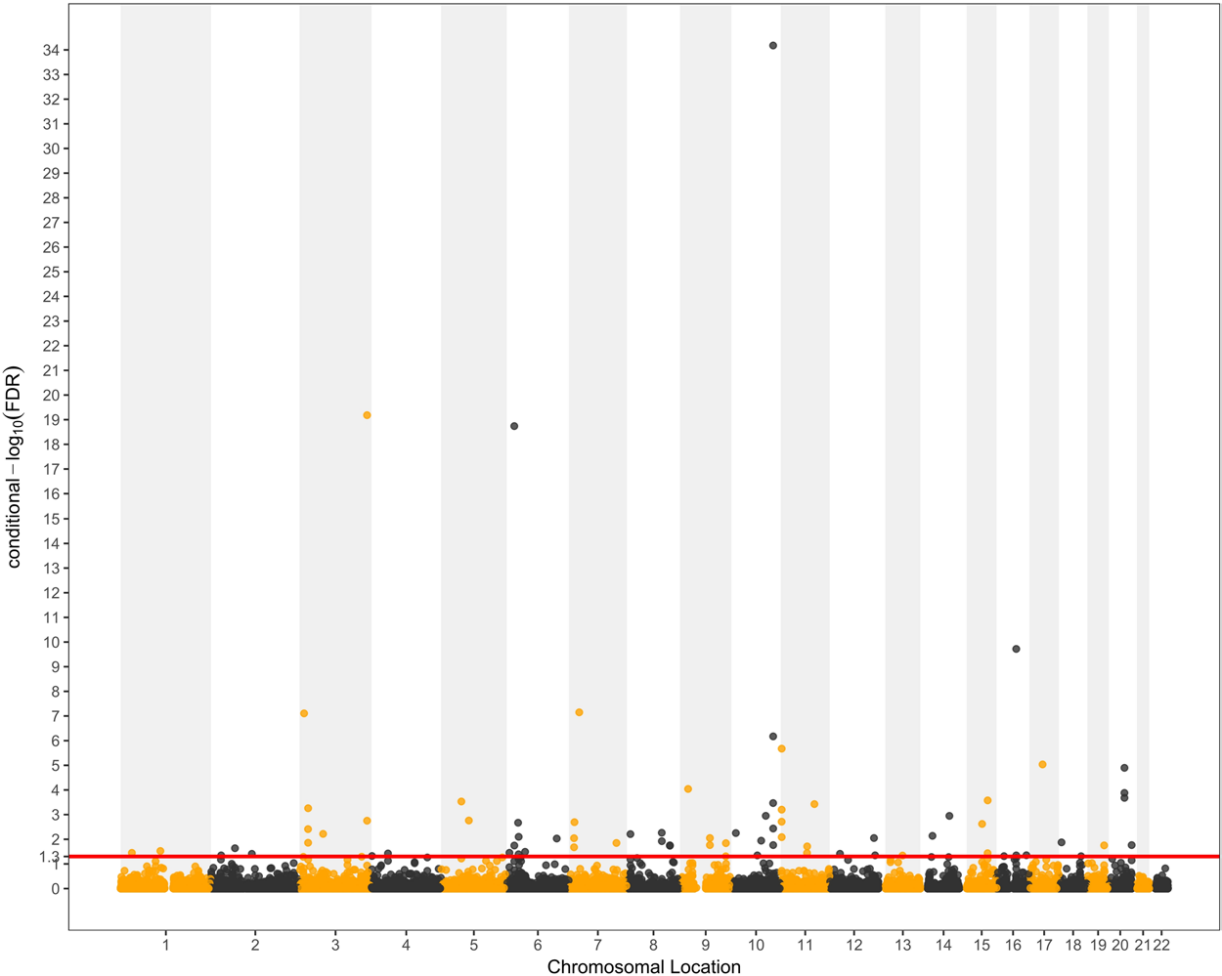
Conditional Manhattan plot of conditional –log_10_ FDR values for T2D given BMI (T2D|BMI). The red line marking the conditional –log_10_ FDR value of 1.3 corresponds to a cFDR < 0.05.

We identified 75 SNPs significantly (cFDR < 0.05) associated with T2D given their association with BMI (Fig. 3 and Table S4), which were located on 20 chromosomes (1–20) and annotated to 89 genes. In the original meta-analysis for T2D GWAS^30^, 38 SNPs had p-values smaller than 1 × 10^−5^ while 12 of them reached the standard genome-wide significance of 5 × 10^−8^. We confirmed 17 SNPs that were reported in the original T2D GWAS analysis^30^ or previous T2D related GWASs^29,35^. Another 18 SNPs that were reported to be associated with T2D-related traits were also confirmed in our analysis^33,36^. The remaining 40 SNPs were not previously reported in the original T2D GWAS^30^ or any other T2D studies, although nine of these SNPs showed high LD (r2 > 0.6) with the T2D-associated SNPs reported previously (Table S5). For the 89 genes these 75 SNPs were annotated to, 42 of these genes were novel and not identified by the original T2D GWAS^30^ or previous T2D-related studies (Table S2). Of the detected loci for T2D, some of the genes were enriched in T2D-related terms such as “pancreas development”, “response to glucose” and “endocrine pancreas development”. GO term enrichment analysis were detailed in Table 1.

### Pleiotropic gene loci for both BMI and T2D

**Figure 4:**
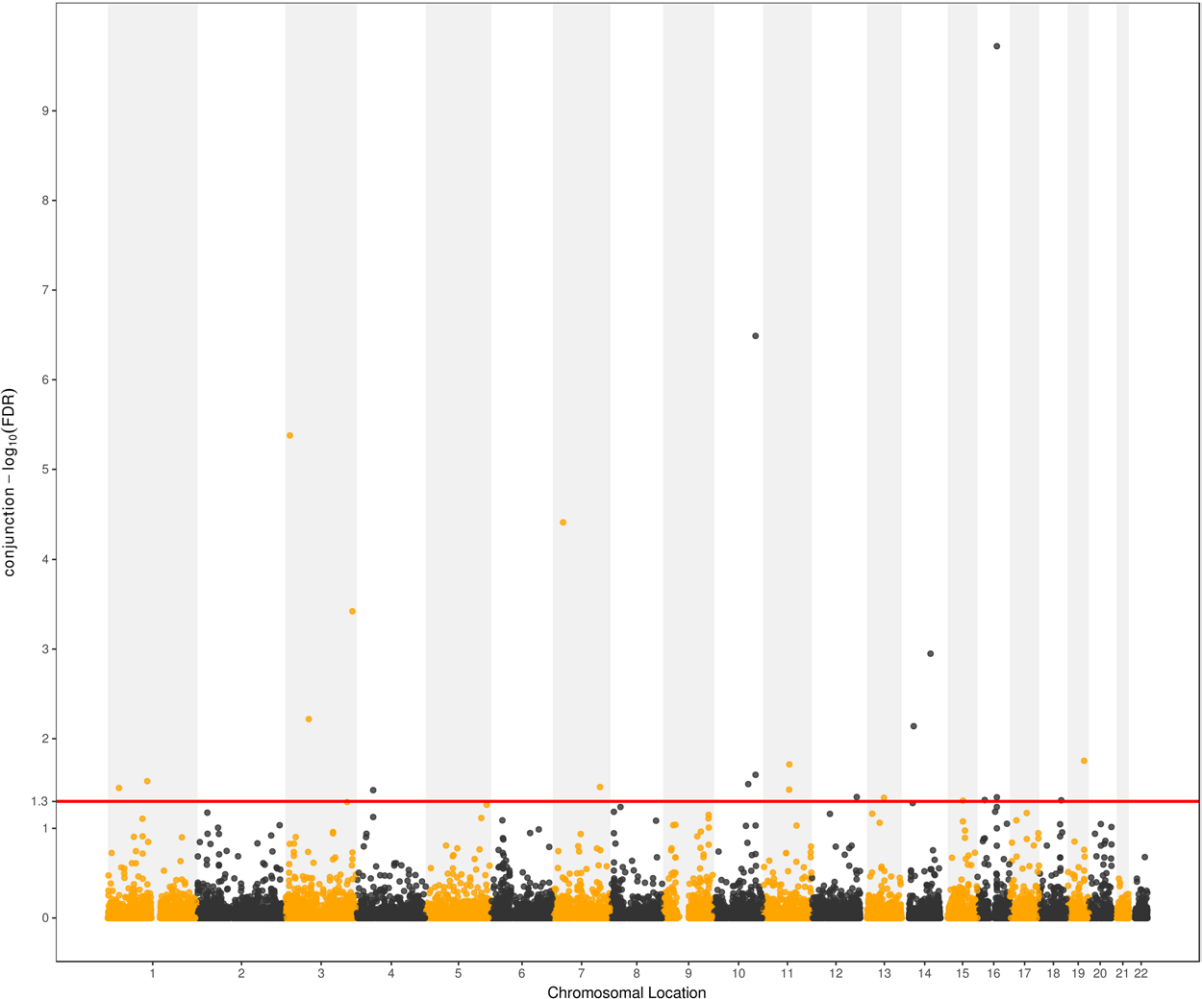
Conjunction Manhattan plot of conjunction –log_10_ FDR values for BMI and T2D. The red line marking the conditional –log_10_ FDR value of 1.3 corresponds to a conjunction FDR < 0.05. The figure shows the genomic locations of pleiotropic loci and further details are provided in Table 2.

The conjunction FDR analysis detected 23 independent pleiotropic loci that were significantly (conjunction FDR < 0.05) associated with both traits (Fig. 4 and Table 2). Of the 23 identified pleiotropic variants, one SNP rs9930506 (*FTO*) reached genome-wide significance in the original BMI and T2D GWASs^30,31^. The SNPs rs7141420 (*NRXN3*), rs1996023 (*GNPDA2* and *GABRG*1), rs16945088 (*FTO*), rs9540493 (*LOC10272396* and *LINC01052*) and rs4238585 (*GPR139* and *GP2*) reached genome-wide significance in only the original BMI GWAS^31^. Six SNPs (rs10787472 (*TCF7L2*), rs2881654 (*PPARG*), rs849135 (*JAZF1*), rs4481184 (*IGF2BP2*), rs1783598 (*FCHSD2*) and rs12245680 (*TCF7L2*)) were reported to be significant for only T2D in the original^30^ or previous T2D GWAS^29^. The two SNPs rs6795735 (*ADAMTS9-AS2*) and rs12454712 (*BCL2*) were previously reported to be associated with both obesity and T2D^29,37,38^. The other five SNPs (rs17584208, rs11979110, rs10898868, rs1996023 and rs4474658) were previously reported to be associated with high density lipoprotein (HDL) and proinsulin^34,39,40^. The final four SNPs were not previously reported in the original BMI and T2D GWASs or GWAS studies for any related traits. For the 30 genes the identified pleiotropic SNPs were annotated to, we found twelve of them (*AKAP6*, *NPAS3*, *PSRC1*, *MYBPHL*, *MIR29A*, *GABRG1*, *ZNF664*, *FAM101A*, *LOC10272396*, *LINC01052*, *GPR139*, and *PUM1*) were not identified by any BMI or T2D related GWASs. For the SNPs that were annotated to these 12 genes, two SNPs were located in the intronic regions of genes *ZNF664* and *PUM1* respectively, and the other five SNPs were all located in intergenic regions (Table 2). Of the detected 23 pleiotropic loci, most of the genes were enriched in BMI and T2D related terms such as “regulation of lipid storage”, “regulation of fat cell proliferation”, “fat cell differentiation”, and “fatty acid transport”. Detailed information of GO term analysis was given in Table 1.

**Table 2:** Conjunction FDR: Pleiotropic Loci in BMI and T2D (cFDR < 0.05).

RSID	ROLE	GENE	CHR	P.valueA	P.valueB	cFDR.AcB	cFDR.BcA	conjunction FDR
**rs9930506**	intronic	FTO	chr16	2.52E–124	1.90E–10	1.01E–123	1.90E–10	1.90E–10
**rs10787472**	intronic	TCF7L2	chr10	3.25E–07	1.30E–36	3.25E–07	6.63E–35	3.25E–07
**rs2881654**	intronic	PPARG	chr3	1.40E–06	3.40E–09	4.19E–06	7.82E–08	4.19E–06
**rs849135**	intronic	JAZF1	chr7	1.45E–05	1.70E–09	3.85E–05	7.08E–08	3.85E–05
**rs4481184**	intronic	IGF2BP2	chr3	0.0002524	4.50E–22	0.0003786	6.55E–20	0.0003786
**rs7141420**	intronic	NRXN3	chr14	8.66E–15	0.00025	4.68E–13	0.001125	0.001125
**rs6795735**	ncRNA_intronic	ADAMTS9–AS2	chr3	2.92E–05	2.00E–04	0.000555	0.00604	0.00604
**rs12895330**	intergenic	AKAP6, NPAS3	chr14	9.72E–05	0.00021	0.0016041	0.007245	0.007245
**rs2334255**	UTR3	GIPR	chr19	0.0008051	0.00034	0.0114503	0.0176422	0.0176422
**rs1783598**	intronic	FCHSD2	chr11	0.0003666	0.00052	0.0064359	0.0193556	0.0193556
**rs12245680**	intronic	TCF7L2	chr10	0.01444	1.10E–09	0.02527	6.70E–07	0.02527
**rs17584208**	intergenic	PSRC1, MYBPHL	chr1	4.58E–06	0.0016	0.000306	0.02976	0.02976
**rs2488071**	intergenic	HHEX, EXOC6	chr10	0.006642	4.70E–06	0.032103	0.0011194	0.032103
**rs11979110**	intergenic	KLF14, MIR29A	chr7	0.00288	9.70E–05	0.03456	0.0140973	0.03456
**rs1473**	intronic	PUM1	chr1	0.0004889	0.00092	0.0114403	0.03542	0.03542
**rs10898868**	intronic	ARAP1	chr11	0.002589	0.00044	0.0370227	0.03608	0.0370227
**rs1996023**	intergenic	GNPDA2, GABRG1	chr4	1.11E–20	0.025	1.93E–17	0.0375	0.0375
**rs825461**	intronic	ZNF664, FAM101A	chr12	0.0003917	0.0013	0.0114376	0.04472	0.04472
**rs16945088**	intronic	FTO	chr16	5.30E–09	0.0072	1.48E–06	0.045	0.045
**rs9540493**	intergenic	LOC10272396, LINC01052	chr13	3.95E–09	0.0057	1.22E–06	0.0456	0.0456
**rs4238585**	intergenic	GPR139, GP2	chr16	1.12E–08	0.0069	3.02E–06	0.0483	0.0483
**rs12454712**	intronic	BCL2	chr18	6.04E–06	0.0034	0.0004955	0.0485714	0.0485714
**rs4474658**	intergenic	C2CD4A, C2CD4B	chr15	0.009024	9.60E–06	0.0489874	0.0023849	0.0489874

**Notes:** P.valueA is the p value of BMI. P.valueB is the p value of T2D.

### Protein-protein interaction network

The 323 identified BMI-associated genes were retrieved from the STRING database. Only 143 genes, including 46 novel genes, were annotated in this database. The 143 genes were clearly enriched in three clusters: *TMEM18*, *PPARG* and *MAP2K5* (Figure S3). Two novel genes *MSRA* and *PDILT*, respectively encoding methionine sulfoxide reductase A and protein disulfide isomerase-like, were directly connected with the *TMEM18* cluster. Another two novel genes, *MED23* and *ANPC4*, respectively encoding mediator complex subunit 23 and anaphase promoting complex subunit 4, were involved in the *PPARG* cluster. Another three novel genes, *MEF2D*, *RASL11A* and *PTPN12*, respectively encoding myocyte enhancer factor 2D, RAS-like, family 11, member A and protein tyrosine phosphatase, were involved in the *MAP2K5* cluster. (Figure S3).

The 89 identified T2D-associated genes were retrieved from the STRING database. Only 37 genes, including 7 novel genes, were annotated in this database. The 37 genes were clearly enriched into three clusters: *HNF4A, MTNR1B* and *TCF7L2* (Figure S4). Three novel genes, *ANXA11*, *BCL2L11* and *NEUROG3*, those respectively encoding Annexin A11, BCL2-like 11 and Neurogenin 3, were involved in the *HNF4A* cluster. Another two novel genes, *NPBWR2* and *PTHLH*, encoding Neuropeptides B/W receptor 2 and Parathyroid hormone-like hormone, were involved in the *MTNR1B* cluster. The other novel gene *MED30* was directly connected with *TCF7L2* cluster (Figure S4)

## Discussion

In our study, two GWASs with summary statistic p values were combined to explore the pleiotropic enrichment of SNPs that are associated with BMI and T2D. Compared to the conventional standard single phenotype GWASs, simultaneously analyzing multiple related traits allows for the increased discovery of trait-associated variants without requiring additional larger datasets for each individual trait. By leveraging the power of two different GWAS datasets from BMI and T2D, we discovered 287 loci for BMI and 75 loci for T2D. Using the standard GWAS significance threshold in the datasets, only 36^31^ were significant for BMI. Most of the genes have not been reported to show borderline significance with BMI, as detailed in Table S2. Adopting the genetic pleiotropy-informed cFDR method, we found 12 additional novel loci associated with both BMI and T2D. These novel findings may enable us to further dissect the overlapping genetic mechanisms between these two related phenotypes. The improved detection of novel susceptibility loci with genetic pleiotropy may lead us to a better understanding of common etiology between disorders and have a significant impact on the clinical treatment and prevention of related complex human diseases.

The cFDR approach was adopted here to account for some of the missing heritability between traits or diseases. This method employs the idea that a variant with significant effects in two associated phenotypes is more likely to be a true effect, and therefore has a higher probability of being detected in multiple independent studies^4,7^. This technique allows for an increase in effective sample size and therefore a subsequent increase in power to detect true associations for more variants with small to moderate effect sizes, which are often ignored in the standard single phenotype GWAS. In addition, the genetic enrichment presented in conditional Q-Q plots conveys that the decreased cFDR value for a given nominal p value greatly increases power to detect true association effects. When initially implementing the cFDR method, Andreassen *et al*.^7^ demonstrated one advantage of this model-free empirical cdf approach is for the avoidance of bias in cFDR estimates from model misspecification. Through a comparison of traditional unconditional FDR and cFDR methods, they found that the latter resulted in an increase of 15–20 times the number of SNPs under the same FDR threshold of 0.05^7^.

Our cFDR analysis identified 23 pleiotropic signals annotated to 30 genes, providing evidence for the close relationship and shared genetic mechanisms between these two traits. These findings are consistent with the evidence from previous studies^25^ that have demonstrated a causal relationship between these two traits. The genes *FTO*, *MC4R* and *TCF7L2* were frequently reported and replicated in previous BMI and T2D related studies^26,30,41^. However, potential confounding factors and biases might coincidently be responsible for some of these associations. For the genes *FTO* and *MC4R*, their respective effects on T2D were found to be modest and previous studies showed that their effects on T2D disappeared after adjustment for BMI^42^. In European populations, *TCF7L2* was not reported as a risk factor for obesity although its effect on T2D risk is modulated by obesity because of the interaction between *TCF7L2* polymorphisms (rs7903146) and BMI status^43^. The implementation of the cFDR method in our study not only furnishes another empirical validation for the cFDR method to successfully detect novel and known disease associated genetic variants, but also shows the possibility of improved discovery of novel susceptibility loci using existing GWAS summary statistics. There were 14 genes (*JAZF1*, *IGF2BP2*, *NRXN3*, *ADAMTS9-AS2*, *GIPR*, *FCHSD2*, *HHEX*, *EXOC6*, *KLF14*, *ARAP1*, *GNPDA2*, *GP2*, *C2CD4A* and *C2CD4B*) that were associated with either BMI or T2D in previous studies but not with both that were detected as pleiotropic loci in this analysis. Furthermore, 12 novel genes are worth noting because no previous study has reported associations with either BMI or T2D for any of them. For the SNPs that were annotated to these 12 genes, two SNPs were located in the intronic regions of genes *ZNF664* and *PUM1* and the other five SNPs were all located in intergenic regions. As examples, we will discuss two of these genes *ZNF664* and *PUM1* for their potential functional relevance and significance.

The SNP rs825461 is located at the intronic region between gene *ZNF664* and *FAM101A*. The *ZNF664* gene encodes a protein named zinc finger protein 664, and one study reported that *ZNF664* was involved in eye development and that the monogenic form may be associated with high risk of myopia^44^. Furthermore, *ZNF664* was previously reported to show suggestive association (P < 1E-4) with adiponectin^45^, a protein involved in many metabolic processes including glucose regulation and fatty acid oxidation^46^. The rs1473 SNP is located at the intronic region of the gene *PUM1*, a member of the PUF family of proteins that contains a sequence-specific RNA binding domain. One study reported that the protein may be involved in the regulation of embryogenesis, and cell development and differentiation^47^. These genes may be involved in certain processes that are significant in the development of obesity and T2D, however future studies are needed to explore the exact mechanisms of those novel genes we identified.

Our study presents several strengths. First, the statistical power is increased through the cFDR method by leveraging two large GWAS datasets, providing an increase in the effective sample size. Although a meta-analysis of the same data would offer a similar gain, the meta-analysis approach only allows for more powerful detection of loci with the same direction of allelic effects in the phenotypes^48^, whereas the cFDR method allows for detection of loci regardless of their effect directions. Second, we consider two traits that are unlikely to be correlated with BMI and T2D, ADHD and MDD, and generate conditional QQ plots with respect to these “control traits.” The “control trait” enrichment analysis provides an alternative way to examine pleiotropic enrichment and provides a baseline that can be used to statistically partially validate the novel findings in our study. We believe that the collider-stratification bias is unlikely in our analysis because, the GWAS datasets have undergone genomic control (GC) and we also carried out LD pruning with r2 > 0.2. In addition, our conditional analysis provides a model-free method to obtain conservative estimation^4,7,8^.

Our study may also have some important limitations. First, we could not provide information about the effect estimates of pleiotropic loci on the phenotypes due to a lack of detailed individual-study-level data. However, we can infer this information from the summary effect sizes in the original GWAS study. This cFDR approach cannot distinguish between vertical and horizontal pleiotropy of the pleiotropic signals, although this question might be partially addressed in future Mendelian Randomization^49,50^ studies. Second, it is likely that some of our cFDR results may be  overestimated due to overlapping samples although the model-free approach is able to neutralize this overestimation of the conservative cFDR estimate^4,7,8^. Alternative approaches may be applied to check whether novel loci could still be identified in order to further confirm novel findings in our study or to furnish an empirical comparison of the relative performance of alternative methods, a topic we wish to pursue in the future with comprehensive theoretical and simulation approaches.

In summary, by incorporating the shared genetic effects of two closely related traits into a conditional analysis framework, we observed significant pleiotropic enrichment between obesity and T2D. We identified several novel pleiotropic loci of potential functional significance for obesity and T2D in our analysis, and the results may provide us with novel insights into the shared genetic influences between these two disorders.

## Materials and Methods

### GWAS Datasets

The dataset for T2D contains association summary statistics for a trans-ethnic T2D GWAS meta-analysis of 26,488 cases and 83,964 controls^30^. Ancestry-specific meta-analyses were previously performed by component datasets from the full set of cohorts, including the DIAbetes Genetics Replication and Meta-analysis (DIAGRAM) Consortium (European descent)^29^, the Asian Genetic Epidemiology Network T2D (AGEN-T2D) Consortium (East Asian descent)^51^, the South Asian T2D (SAT2D) Consortium (south Asian descent)^52^, and the Mexican American T2D (MAT2D) Consortium (Mexican and Mexican American descent)^53^. Further details of the samples and methods employed within each ancestry group are presented in the corresponding consortium papers^29,51–53^. Briefly, various genotyping products were applied in the individual study’s assay processes, with appropriate sample and SNP quality control (QC). Genotype imputation was conducted within each GWAS dataset using Phase II/III HapMap as the reference panels. Each SNP with MAF > 1% (except MAF > 5% in MAT2D GWAS due to a smaller sample size) and passing QC was analyzed for association with T2D using an adjusted additive model. Association summary statistics of each ancestry-specific meta-analysis were combined using a fixed-effect inverse-variance weighted meta-analysis. Genomic control (GC) was carried at the individual study level, after ethnicity-specific meta-analysis, and finally after trans-ethnic meta-analysis^30^.

The GIANT dataset for BMI contains association summary statistics for the GWAS and Metabochips meta-analysis of 339,224 individuals of various ancestries, including 322,154 individuals of European descent and 17,072 individuals of African-American and Hispanic descent^31^. The data contains the summary p-values from meta-analysis after correction for inflation due to potential population admixture. Two rounds of GC were separately applied both at the cohort level and after meta-analysis^31^.

### Data Preparation

Before the implementation of the cFDR method, several preparation steps were performed. First, we checked the European Ancestry cohorts for overlapping samples included in these two datasets (Table S1). Next, we combined the common SNPs included in these two datasets. Then we applied a linkage disequilibrium (LD) based SNP pruning method^7,8^ to remove large correlations between pairs of variants. The SNP pruning method begins using a window of 50 SNPs where LD is calculated between each pair of SNPs. The minor allele frequency (MAF) is the basis for the SNP pruning, where for pairs with r^2^ > 0.2 we removed the SNP with smaller MAF. Following this initial removal of SNPs, the window slides 5 SNPs forward and the process is repeated until there are no pairs of SNPs that are high in LD. The dataset was pruned using the HapMap 3 genotypes as a reference panel. Last, we performed gene annotation for the final set of 123,804 variants that were included in the analyses.

GC corrections were used in the GWASs to ensure that the variance estimates for each SNP are not inflated due to population structure and cryptic relatedness^54^. Both of the original datasets^30,31^ we adopted in our study applied GC at the individual study level and again after meta-analysis, hence there was no need for us to reapply the GC in this analysis.

### Statistical analysis

The cFDR approach is well-established now, which has been widely applied by many other groups^4,7,8,55,56^ and our group^12–14^. We briefly summarized this cFDR approach as follows: after the data preparation processing, we computed the conditional empirical cumulative distribution functions (cdfs) of the corrected p-values for the x axis in conditional QQ plot. Empirical cdfs for BMI SNP p-values were conditioned on nominal p-values in T2D, and *vice versa*. For each nominal p-value, an estimate of the cFDR was obtained from the conditional empirical cdfs. Using this cFDR approach, we obtained two cFDR tables–cFDR result for BMI conditioned on T2D and *vice versa*. Using these tables we identified loci associated with BMI and T2D (cFDR < 0.05), respectively. Then a conjunction method was used to find SNPs significantly associated with both BMI and T2D. Specifically, we took the maximum of those two cFDR values above as our conjunction FDR.

### Conditional QQ and enrichment plots for assessing pleiotropic enrichment

As an intuitive illustration, we presented conditional Q-Q plots to graphically assess the pleiotropic enrichment of SNPs of the principal phenotype successively conditioning on various strengths of associations with the conditional phenotype. We plotted the QQ curve for the quantiles of nominal ${{\rm{\mbox{--}}}{\rm{log}}}_{10}({\rm{p}})$-values obtained from GWAS summary statistics for association of the subset of SNPs that are below each significance threshold in the conditional trait. The nominal ${{\rm{\mbox{--}}}{\rm{log}}}_{10}({\rm{p}})$-values were plotted on the y-axis and the empirical quantiles (empirical cumulative distribution functions (cdfs)) of the nominal p-values were plotted on the x-axis. Under the global null hypothesis, the theoretical distribution of p-values is expected to lie approximately on the diagonal line of the Q-Q plots. Enrichment of genetic associations is indicated as a leftward deflection from the null line as the principal phenotype is successively conditioned on increasing strength of associations with the conditional phenotype. The degree of deflection between curves provides important information about the degree of pleiotropy between the two phenotypes. Larger deflections are considered to represent a greater enrichment of pleiotropic genes between the two phenotypes.

For the associated phenotypes BMI and T2D, pleiotropic “enrichment” of BMI with T2D exists if the proportion of SNPs or genes associated with BMI increases as a function of increased association with T2D. To confirm the pleiotropic enrichment effect, we presented fold-enrichment plots of nominal ${{\rm{\mbox{--}}}{\rm{log}}}_{10}({\rm{p}})$ values for BMI SNPs below the standard GWAS threshold of p < 5 × 10^−8^ and for subsets of SNPs determined by the significance of their association with T2D and *vice versa*. As the p values of the conditional phenotypes become more significant, lower upward shift from the null line will persist.

In order to check the pleiotropic enrichment and provide a baseline that can be used to confirm novel findings, we also generated conditional QQ plots for two control traits that are unlikely to be correlated with BMI and T2D, ADHD and MDD.

### Conditional Manhattan plots for localizing genetic variants

To demonstrate the localization of the SNPs associated with BMI conditional on their significance on T2D, and the reverse, we present conditional Manhattan plots. The plots present the relationship between all SNPs within an LD block and their chromosomal locations. The 22 chromosomal locations are plotted on the x-axis, and the ${{\rm{\mbox{--}}}{\rm{log}}}_{10}({\rm{FDR}})$ BMI values conditional on T2D are plotted on the y-axis and *vice versa* for T2D. Any SNP with a ${{\rm{\mbox{--}}}{\rm{log}}}_{10}({\rm{FDR}})$ value greater than 1.3 (FDR < 0.05) was deemed to be significantly associated with the principal phenotype. We also present a conjunction Manhattan plot to demonstrate the locations of the common pleiotropic genetic variants associated with both phenotypes.

### Functional annotation and gene enrichment analysis

In order to evaluate the biological functions of the individual trait associated loci identified by cFDR and pleiotropic loci identified by conjunction FDR, we performed functional annotation and gene enrichment analysis using the gene ontology (GO) terms database (http://geneontology.org/)^57^. All significant genes identified by cFDR and conjunction cFDR in our study were annotated and characterized based on three main categories: biological processes, cellular component and molecular functions. This analysis provided comprehensive biological information, allowing us to partially validate our findings by determining specific genes that are enriched in T2D- and obesity-related GO terms.

### Protein-protein interaction network

In order to detect interactions and associations of the BMI-associated and T2D-associated genes respectively, protein-protein interaction analyses were conducted by searching the Search Tool for the Retrieval of Interacting Genes/Proteins (STRING) database (http://string-db.org/). The STRING database comprises known and predicted associations from curated databases or high-throughput experiments, and also with other associations derived from text mining, co-expression, and protein homology^58^.

## Electronic supplementary material

supplementary files
