# Supplementary material for: Novel Common Variants Associated with Obesity and Type 2 Diabetes Detected Using a cFDR Method
Source: Sci Rep. 2017 Nov 27;7:16397. doi: 10.1038/s41598-017-16722-6 (PMC5703959; doi:10.1038/s41598-017-16722-6)
Supplement: Supplementary file 1 — supplementary files [file 41598_2017_16722_MOESM1_ESM.pdf]

Novel Common Variants Associated with Obesity and Type 2 Diabetes Detected Using a cFDR Method

Qiang Zhang[1], Ke-Hao Wu[2], Jing-Yang He[1], Yong Zeng [2,4], Jonathan Greenbaum[2], Xin Xia[1], Hui-Min Liu[1], Wan-Qiang Lv[1], Xu Lin[3], Wei-Dong Zhang[1], Yuan-Lin Xi[1], Xue-Zhong Shi[1], Chang-Qing Sun[1*], Hong-Wen Deng[1,2*]

[1]College of Public Health, Zhengzhou University, Zhengzhou, NO.100 Kexue Road, High-Tech Development Zone Of States, P.R.C

[2]Center for Bioinformatics and Genomics, School of Public Health and Tropical Medicine, Tulane University, New Orleans, LA 70112, USA

[3]Department of Endocrinology and Metabolism, the Third Affiliated Hospital of Southern Medical University, Guang Zhou, P.R.C

[4]College of Sciences, Beijing Jiao Tong University, Beijing, China

***Corresponding author**

Chang-Qing Sun, Prof.

Department of Social Medicine and Health Management

College of Public Health, Zhengzhou University100 Kexue Avenue

Zhengzhou, 450001, Henan, PR China

Phone: +86 371 67781926

Fax: +86 371 67781919

E-mail: suncq@zzu.edu.cn

Hong-Wen Deng, Prof.

Department of Epidemiology and Biostatistics

College of Public Health, Zhengzhou University100 Kexue Avenue

Zhengzhou, 450001, Henan, PR China

Tulane Center of Bioinformatics and Genomics

Department of Biostatistics and Data Science

Tulane University School of Public Health and Tropical Medicine

New Orleans, LA 70112, USA

Email: hdeng2@tulane.edu

Supplemental Information

Table S1. Overlapped individuals between BMI and T2D in European descendants.

Table S2. Conditional FDR value for BMI given the T2D ($cFDR < 0.05$).

Table S3. SNPs in high LD ($R^2 > 0.6$) with BMI-associated loci

Table S4. Conditional FDR value for T2D given the BMI ($cFDR < 0.05$).

Table S5. SNPs in high LD ($R^2 > 0.6$) with T2D-associated loci

Figure S1. Stratified QQ plots. Upper Panel: Stratified QQ plots of nominal versus empirical $-\log_{10}$ p-values in (left) BMI as a function of significance of the association with ADHD, and in (right) ADHD as a function of significance of the association with BMI. Lower Panel: Stratified QQ plots of nominal versus empirical $-\log_{10}$ p-values in (left) BMI as a function of significance of the association with MDD, and in (right) MDD as a function of significance of the association with BMI.

Figure S2. Stratified QQ plots. Upper Panel: Stratified QQ plots of nominal versus empirical $-\log_{10}$ p-values in (left) T2D as a function of significance of the association with ADHD, and in (right) ADHD as a function of significance of the association with T2D. Lower Panel: Stratified QQ plots of nominal versus empirical $-\log_{10}$ p-values in (left) T2D as a function of significance of the association with MDD, and in (right) MDD as a function of significance of the association with T2D.

Figure S3. Protein-protein interactions between BMI-associated genes.

Figure S4. Protein-protein interactions between T2D-associated genes.

Table S1: Overlapped individuals between T2D and BMI in European descendants

study	Full Name	T2D	BMI
**ARIC**	Atherosclerosis Risk in Communities Study	cases,775;controls,7,159	8,108
**deCODE**	deCODE genetics sample set	cases,1,465;controls,23,194	26,799
**PROMIS**	Pakistan Risk of Myocardial Infarction Study	cases,2,361;controls,6,817	3,615
**DGI**	Diabetes Genetics Initiative of Broad Institute of Harvard and MIT, Lund University, and Novartis Institutes of BioMedical Research	cases,1,022;controls,1,075	cases,1,317;controls,1,088
**FUSION**	Finland-United States Investigation of NIDDM Genetics	cases,1,161;controls,1,174	cases,1,092;controls,1,171
**NHS**	The Nurses' Health Study	cases,1,467;controls,1,754	2,265
**T2D_WTCCC**	WTCCC Type 2 Diabetes cases	cases,1,924	cases,1,903

Table S2 Conditional FDR value for BMI given the T2D (cFDR < 0.05).

RSID	ROLE	GENE	CHR	SNP type	Gene type	P.valueA	cFDR.AcB
**rs9930506**	intronic	FTO	chr16	Confirmed	Confirmed	2.52E-124	1.01E-123
**rs7531118**	intergenic	NEGR1, LINC01360	chr1	Confirmed	Confirmed	1.88E-28	5.07E-24
**rs1996023**	intergenic	GNPDA2, GABRG1	chr4	Confirmed	Confirmed, confirmed	1.11E-20	1.93E-17
**rs11672660**	intronic	GIPR	chr19	Confirmed	Confirmed	7.91E-19	4.31E-15
**rs7141420**	intronic	NRXN3	chr14	Confirmed	Confirmed	8.66E-15	4.68E-13
**rs1385912**	intronic	NEGR1	chr1	Confirmed	Confirmed	5.79E-17	7.00E-13
**rs1031477**	intronic	NCKAP5L	chr12	Confirmed	Confirmed	4.87E-16	7.59E-13
**rs2635727**	intergenic	TFAP2B, PKHD1	chr6	Confirmed	Confirmed, confirmed	1.35E-15	1.25E-11
**rs1849338**	intergenic	GNPDA2, GABRG1	chr4	Confirmed	Confirmed, confirmed	5.09E-14	4.70E-11
**rs7553348**	intronic	FPGT-TNNI3K, TNNI3K	chr1	Confirmed	Confirmed, confirmed	4.40E-15	5.56E-11
**rs2902210**	intronic	SEC16B	chr1	Confirmed	Confirmed	3.37E-14	2.10E-10
**rs12429545**	intergenic	LINC01065, LINC00558	chr13	Confirmed	Confirmed, confirmed	3.15E-13	2.90E-10
**rs12956917**	intergenic	PMAIP1, MC4R	chr18	Confirmed	Confirmed, confirmed	2.09E-13	1.73E-09
**rs3922668**	intronic	SPNS1	chr16	Confirmed	Confirmed	7.95E-13	7.08E-09

**rs11125884**	ncRNA_intronic	DNAJC27-AS1	chr2	Confirmed	Confirmed	1.74E-12	8.98E-09
**rs7939420**	intronic	AGBL2	chr11	Confirmed	Confirmed	2.22E-12	9.91E-09
**rs2060604**	intergenic	HNF4G, LINC01111	chr8	Confirmed	Confirmed, confirmed	9.46E-12	4.12E-08
**rs6548242**	intergenic	FAM150B, TMEM18	chr2	Confirmed	Confirmed, confirmed	6.14E-11	9.28E-08
**rs752720**	intergenic	PMAIP1, MC4R	chr18	Confirmed	Confirmed, confirmed	6.63E-11	9.81E-08
**rs10787472**	intronic	TCF7L2	chr10	T2D (26551672)	Confirmed	3.25E-07	3.25E-07
**rs4704220**	intronic	COL4A3BP	chr5	LDL (20686565)	Confirmed	4.90E-10	8.96E-07
**rs9540493**	intergenic	LOC102723968, LINC01052	chr13	Confirmed	Confirmed, confirmed	3.95E-09	1.22E-06
**rs16945088**	intronic	FTO	chr16	Confirmed	Confirmed	5.30E-09	1.48E-06
**rs6658131**	intergenic	LOC101928241, PTBP2	chr1	Confirmed	Confirmed, confirmed	1.97E-09	1.52E-06
**rs1032524**	ncRNA_intronic	IPO9-AS1	chr1	Confirmed	Confirmed	5.64E-10	2.01E-06
**rs4238585**	intergenic	GPR139, GP2	chr16	Confirmed	Confirmed, confirmed	1.12E-08	3.02E-06
**rs2881654**	intronic	PPARG	chr3	Fasting insulin adjusted for BMI (22885924)	Confirmed	1.40E-06	4.19E-06
**rs522367**	intronic	SEC16B	chr1	Confirmed	Confirmed	1.97E-09	5.82E-06
**rs815611**	intergenic	MFAP3, GALNT10	chr5	Confirmed	Confirmed, confirmed	2.36E-08	8.50E-06
**rs6713510**	ncRNA_intronic	LOC646736	chr2	Confirmed	Confirmed	1.97E-08	8.62E-06
**rs2038645**	intergenic	LOC101928241,	chr1	Confirmed	Novel, confirmed	1.01E-08	9.37E-06

		PTBP2					
**rs17217144**	intronic	FTO	chr16	Confirmed	Confirmed	4.10E-08	9.96E-06
**rs12986742**	ncRNA_intronic	LINC01122	chr2	Confirmed	Confirmed	8.92E-09	1.13E-05
**rs9958194**	intergenic	PMAIP1, MC4R	chr18	Confirmed	Confirmed, confirmed	3.02E-08	1.15E-05
**rs1275986**	intergenic	CIB4, KCNK3	chr2	Confirmed	Novel, confirmed	4.88E-08	1.28E-05
**rs1780050**	intronic	NEXN	chr1	Confirmed	Confirmed	2.71E-08	2.01E-05
**rs11755393**	exonic	UHRF1BP1	chr6	Confirmed	Confirmed	2.43E-08	2.42E-05
**rs891386**	intronic	C18orf8	chr18	Confirmed	Confirmed	5.20E-08	2.90E-05
**rs16952522**	intronic	FTO	chr16	Confirmed	Confirmed	8.47E-08	2.94E-05
**rs849135**	intronic	JAZF1	chr7	Height (23754948)	Confirmed	1.45E-05	3.85E-05
**rs2235569**	intronic	C6orf106	chr6	**LD**	Confirmed	9.64E-08	6.26E-05
**rs757318**	intronic	CRTC1	chr19	Confirmed	Confirmed	3.18E-08	6.46E-05
**rs17243334**	intronic	MAP2K5	chr15	**LD**	Confirmed	3.09E-07	9.45E-05
**rs6990042**	intronic	SGCZ	chr8	Novel	Confirmed	4.48E-07	0.000165225
**rs7503807**	intronic	RPTOR	chr17	**LD**	Confirmed	5.73E-07	0.00017371
**rs185350**	downstream	KCTD15	chr19	BMI (23754948)	Confirmed	7.63E-08	0.000248708
**rs4949662**	intronic	ZZZ3	chr1	**LD**	Obesity (23563607)	4.98E-07	0.000278265
**rs1222069**	intergenic	LOC102723661, LOC101928241	chr1	Confirmed	Confirmed, novel	1.64E-06	0.000282799
**rs7197983**	intronic	FTO	chr16	Novel	Confirmed	2.05E-07	0.000292654
**rs2335418**	intergenic	ANKRD31, HMGCR	chr5	LDL/total cholesterol (20686565)	Confirmed, confirmed	6.88E-07	0.000303523
**rs17584208**	intergenic	PSRC1, MYBPHL	chr1	LDL/total cholesterol (20686565)	Confirmed, novel	4.58E-06	0.000306011

**rs258496**	intergenic	POC5, SV2C	chr5	LDL/total cholesterol (24097068)	Confirmed, novel	4.88E-07	0.000357566
**rs6720683**	intergenic	FAM150B, TMEM18	chr2	Novel	Novel, confirmed	2.33E-07	0.000368699
**rs1440983**	intergenic	PRKD1, G2E3	chr14	BMI (23754948)	Confirmed, novel	3.56E-06	0.000374722
**rs11079813**	intronic	SKAP1	chr17	Novel	Confirmed	2.95E-06	0.000376817
**rs4481184**	intronic	IGF2BP2	chr3	T2D (24509480)	Confirmed	0.0002524	0.0003786
**rs11632574**	intronic	MAP2K5	chr15	**LD**	Confirmed	1.67E-06	0.000414023
**rs10840103**	intergenic	TRIM66, RPL27A	chr11	**LD**	Confirmed, confirmed	1.74E-07	0.000427491
**rs7239883**	ncRNA_intronic	LINC00907	chr18	Confirmed	Confirmed	3.14E-07	0.000435037
**rs12454712**	intronic	BCL2	chr18	Waist hip ratio adjusted for BMI (25673412)	Confirmed	6.04E-06	0.000495526
**rs6795735**	ncRNA_intronic	ADAMTS9-AS 2	chr3	Waist hip ratio adjusted for BMI (25673412)	Confirmed	2.92E-05	0.00055499
**rs11688816**	intronic	EHBP1	chr2	Confirmed	Confirmed	3.80E-07	0.000573136
**rs1861569**	intergenic	MIR5095, CBLN1	chr16	**LD**	Novel, confirmed	5.67E-06	0.000575201
**rs6864049**	intergenic	ZNF608, LOC101927421	chr5	Waist circumference (25673412)	Confirmed, confirmed	2.12E-07	0.000624587
**rs17110049**	intergenic	STXBP6, NOVA1	chr14	Novel	Confirmed, confirmed	3.16E-07	0.00062492
**rs405509**	upstream	APOE	chr19	LDL cholesterol	Confirmed	2.65E-07	0.00067586

				(20686565)			
**rs2270204**	intronic	SWI5	chr9	Novel	Confirmed	3.22E-07	0.000726505
**rs740158**	intergenic	LOC101927243, PTPN12	chr7	Novel	Novel, novel	3.98E-06	0.000738203
**rs1549979**	intronic	CADM2	chr3	**LD**	Confirmed	2.71E-07	0.000749461
**rs1979755**	intronic	KCNG3	chr2	Novel	Confirmed	4.33E-07	0.000950734
**rs4807191**	intronic	BTBD2	chr19	Novel	Novel	7.47E-07	0.000955929
**rs9784046**	intergenic	FAM150B, TMEM18	chr2	**LD**	Novel, confirmed	4.76E-07	0.000983564
**rs10499694**	intronic	DDC	chr7	Novel	Confirmed	4.72E-07	0.000987047
**rs1546924**	intronic	FAM212B	chr1	Novel	Confirmed	4.93E-06	0.000989891
**rs1561277**	intronic	ZRANB3	chr2	Height (25282103)	Height (25282103)	7.85E-07	0.001078547
**rs7433760**	intronic	ETV5	chr3	Novel	Confirmed	1.02E-06	0.001090877
**rs12681744**	intergenic	HNF4G, LINC01111	chr8	Novel	Confirmed, hip circumference (25673412)	1.01E-06	0.001127745
**rs7633265**	intronic	ACAP2	chr3	Novel	Confirmed	6.62E-07	0.001299579
**rs13432055**	intronic	CCDC85A	chr2	Novel	Novel	1.14E-05	0.001313946
**rs998584**	intergenic	, LINC01512	chr6	Waist hip ratio (25673412)	Confirmed, novel	2.89E-06	0.001315945
**rs227761**	intronic	IQCK	chr16	Novel	Confirmed	3.81E-06	0.001455138
**rs4290163**	intergenic	CYP17A1, BORCS7	chr10	Hip circumference (25673412)	Novel, novel	7.35E-07	0.00151012
**rs12895330**	intergenic	AKAP6, NPAS3	chr14	Novel	Confirmed, confirmed	9.72E-05	0.00160413
**rs9381901**	intergenic	TFAP2D, TFAP2B	chr6	**LD**	Confirmed, confirmed	1.77E-06	0.001629138

**rs12044597**	intronic	NADK	chr1	Novel	Novel	1.01E-06	0.001646721
**rs9408902**	intergenic	ASTN2, LOC101928797	chr9	**LD**	Novel, confirmed	3.06E-06	0.001713757
**rs3930017**	intergenic	PMS2P9, CCDC146	chr7	Novel	Novel, novel	9.35E-07	0.001744005
**rs4937993**	intronic	NCAM1	chr11	Novel	Confirmed	4.70E-06	0.001846511
**rs2444217**	intronic	ADCY9	chr16	Novel	Confirmed	1.92E-06	0.001940733
**rs6730157**	intronic	RAB3GAP1	chr2	Height (25282103)	Confirmed	2.04E-06	0.002005948
**rs12127789**	intronic	NEGR1	chr1	**LD**	Confirmed	1.54E-05	0.00203588
**rs7232886**	intergenic	PMAIP1, MC4R	chr18	**LD**	Confirmed, confirmed	1.53E-06	0.002068537
**rs4988235**	intronic	MCM6	chr2	Hip circumference (25673412)	Confirmed	2.14E-06	0.002069618
**rs7551794**	intergenic	PTBP2, DPYD	chr1	Novel	Confirmed, confirmed	3.81E-06	0.002080004
**rs12447481**	intronic	FTO	chr16	Novel	Confirmed	1.30E-05	0.002233689
**rs6465468**	UTR3	ASB4	chr7	Confirmed	Confirmed	2.44E-06	0.00240137
**rs4809401**	exonic	NPBWR2	chr20	Novel	Confirmed	7.51E-06	0.002417407
**rs4771946**	intronic	HS6ST3	chr13	**LD**	Confirmed	2.57E-06	0.00256469
**rs751008**	intronic	DOCK1	chr10	Novel	Confirmed	1.64E-06	0.002861018
**rs2685230**	intergenic	FAM150B, TMEM18	chr2	Novel	Novel, confirmed	4.88E-06	0.00286647
**rs4304326**	intergenic	DUSP26, LINC01288	chr8	Novel	Confirmed, confirmed	9.32E-06	0.002929285
**rs2074120**	intronic	CALCR	chr7	Novel	Confirmed	8.81E-06	0.002932137
**rs4667682**	intergenic	TLK1,	chr2	Novel	Novel, confirmed	3.43E-06	0.0030852

		METTL8					
**rs9659092**	intronic	AGBL4	chr1	**LD**	Confirmed	1.86E-06	0.003136918
**rs7748777**	intergenic	TREM2, TREML2	chr6	Novel	Novel, novel	1.27E-05	0.003178313
**rs7228347**	intergenic	MC4R, CDH20	chr18	**LD**	Confirmed, novel	9.41E-06	0.003254209
**rs10971712**	intronic	UBE2R2	chr9	Novel	Confirmed	1.51E-05	0.003293576
**rs12598706**	intronic	PDILT	chr16	Novel	Novel	4.34E-06	0.003310199
**rs17700028**	intergenic	PMAIP1, MC4R	chr18	Novel	Confirmed, confirmed	2.63E-05	0.003315913
**rs2665357**	intronic	SLC22A3	chr6	Triglycerides (24097068)	Confirmed	2.17E-05	0.003381668
**rs10850838**	intergenic	MIR4497, GLTP	chr12	Novel	Novel, novel	2.49E-05	0.003760116
**rs11786541**	intergenic	MIR124-1, MSRA	chr8	Novel	Novel, novel	6.20E-05	0.003802053
**rs1225051**	intronic	CPNE4	chr3	Novel	Novel	5.23E-06	0.003898182
**rs1433075**	intronic	ZNF536	chr19	Novel	Confirmed	1.53E-05	0.00418506
**rs17594321**	intronic	ICA1L	chr2	Novel	Novel	4.10E-05	0.00433728
**rs2150855**	intronic	LINGO2	chr9	Novel	Confirmed	3.52E-06	0.004399431
**rs10934987**	intronic	CPNE4	chr3	Novel	Confirmed	5.03E-05	0.004563173
**rs17049738**	ncRNA_intronic	LINC01122	chr2	Novel	Confirmed	4.36E-05	0.004600947
**rs9357506**	intronic	RCAN2	chr6	Novel	Confirmed	6.80E-06	0.004685473
**rs17326595**	intergenic	KCTD15, LSM14A	chr19	**LD**	Confirmed, novel	3.44E-06	0.004839931
**rs7586043**	intergenic	LINC01320,	chr2	Novel	Novel, confirmed	3.94E-05	0.005117885

		LOC100288911					
**rs2280018**	intronic	PDXDC1	chr16	Novel	Confirmed	4.70E-05	0.005435586
**rs9639027**	upstream	ZNF12	chr7	Novel	Confirmed	4.50E-06	0.005515095
**rs10069222**	intronic	SLIT3	chr5	Novel	Novel	0.0001328	0.006049778
**rs718947**	intronic	CTBP2	chr10	Novel	Confirmed	6.51E-06	0.006061564
**rs11064524**	intronic	WNK1	chr12	Novel	Confirmed	5.16E-06	0.0063081
**rs3814878**	UTR5	FAM57B	chr16	**LD**	Novel	2.80E-05	0.0063371
**rs1783598**	intronic	FCHSD2	chr11	Fasting glucose (22885924)	Fasting glucose (22885924)	0.0003666	0.006435867
**rs2033530**	downstream	TDRG1	chr6	Novel	Confirmed	8.22E-06	0.00650742
**rs12465802**	intronic	R3HDM1	chr2	Height (25282103)	Height (25282103)	9.85E-06	0.006633446
**rs11066301**	intronic	PTPN11	chr12	Total cholesterol (20686565)	Total cholesterol (20686565)	6.32E-06	0.006673692
**rs6723710**	ncRNA_intronic	LINC01122	chr2	Novel	Confirmed	1.00E-05	0.006707388
**rs9296115**	intronic	PACSIN1	chr6	Novel	Novel	6.80E-06	0.007031531
**rs11022762**	intronic	ARNTL	chr11	Novel	Confirmed	1.95E-05	0.007220381
**rs285556**	intronic	PDZRN4	chr12	Novel	Confirmed	6.80E-06	0.007611221
**rs10760450**	UTR3	LMX1B	chr9	Novel	Confirmed	7.69E-06	0.007660047
**rs3922649**	intergenic	LOC101928778, SEC16B	chr1	Novel	Confirmed, confirmed	6.72E-06	0.00766394
**rs1899689**	intronic	CADPS2	chr7	Novel	Novel	6.68E-05	0.007969874
**rs3756784**	upstream	MED23	chr6	Novel	Novel	0.0001189	0.008434906
**rs7970953**	intronic	SOX5	chr12	Novel	Confirmed	1.52E-05	0.00878693
**rs836964**	intronic	FAIM2	chr12	Novel	Confirmed	8.05E-06	0.008812782
**rs4686734**	intergenic	ETV5, DGKG	chr3	Novel	Confirmed, confirmed	2.96E-05	0.009353304

**rs1609761**	intronic	REXO1	chr19	Novel	Novel	3.62E-05	0.009428682
**rs7594432**	intronic	DNMT3A	chr2	Height (25282103)	Confirmed	4.62E-05	0.009457659
**rs823130**	intronic	NUCKS1	chr1	Novel	Novel	0.0001112	0.009567833
**rs763310**	intronic	KDM1A	chr1	Novel	Novel	1.04E-05	0.009579896
**rs8087237**	intronic	PTPN2	chr18	Novel	Confirmed	8.37E-06	0.009581891
**rs7239114**	intergenic	ZBTB7C, CTIF	chr18	Novel	Novel, novel	3.58E-05	0.009653323
**rs11625620**	intronic	NRXN3	chr14	Novel	Confirmed	1.03E-05	0.009673169
**rs3752904**	exonic	ECE2	chr3	Novel	Novel	4.81E-05	0.009763351
**rs2225203**	intergenic	LINC01065, LINC00558	chr13	Novel	Novel, novel	1.47E-05	0.009920883
**rs8048267**	intronic	ZFHX3	chr16	Novel	Confirmed	6.15E-05	0.009988875
**rs10913472**	intronic	SEC16B	chr1	Novel	Confirmed	1.08E-05	0.010480014
**rs10774625**	intronic	ATXN2	chr12	Novel	Confirmed	1.28E-05	0.010884146
**rs10932150**	exonic	ZDBF2	chr2	Novel	Confirmed	8.98E-05	0.011110275
**rs17819063**	intronic	FTO	chr16	Novel	Confirmed	1.07E-05	0.011420296
**rs825461**	intronic	ZNF664, FAM101A	chr12	Waist hip ratio adjusted for BMI in females greater than 50 years of age (26426971)	Confirmed, confirmed	0.0003917	0.01143764
**rs6510001**	intergenic	URI1, ZNF536	chr19	Novel	Novel, confirmed	2.73E-05	0.011439555
**rs1473**	intronic	PUM1	chr1	Novel	Novel	0.0004889	0.01144026
**rs2334255**	UTR3	GIPR	chr19	Novel	Confirmed	0.0008051	0.011450311
**rs11925138**	intronic	CPNE4	chr3	Novel	Confirmed	7.69E-05	0.011637533
**rs882386**	intronic	DMXL2	chr15	Novel	Confirmed	2.14E-05	0.011834154

**rs4717540**	intronic	AUTS2	chr7	Novel	Confirmed	0.0001241	0.012437147
**rs17783165**	intronic	CDH7	chr18	Novel	Confirmed	0.0001871	0.0127228
**rs17211262**	intergenic	MMD, TMEM100	chr17	Novel	Confirmed, novel	7.06E-05	0.012791893
**rs4796221**	intergenic	TBC1D3B, ZNHIT3	chr17	Novel	Novel, novel	7.06E-05	0.013475342
**rs2161767**	intergenic	MIR1302-4, CREB1	chr2	Novel	Novel, confirmed	1.47E-05	0.013701993
**rs4980550**	intergenic	MYEOV, LINC01488	chr11	Novel	Confirmed, novel	1.67E-05	0.013717911
**rs329655**	intergenic	IGSF9B, LOC100128239	chr11	Novel	Novel, novel	5.71E-05	0.013987777
**rs2012284**	intronic	ANAPC4	chr4	Novel	Novel	0.0002204	0.014798286
**rs7161194**	downstream	MIR377	chr14	Novel	Novel	3.02E-05	0.014800976
**rs17644631**	intergenic	LOC101927285, MIR4432HG	chr2	Novel	Confirmed, novel	0.000131	0.0148816
**rs16969473**	intronic	GPR139	chr16	Novel	Confirmed	3.36E-05	0.014918456
**rs984677**	intergenic	CASC20, BMP2	chr20	Height (23754948)	Confirmed, confirmed	5.63E-05	0.015038298
**rs2965189**	intronic	GATAD2A	chr19	Total cholesterol (24097068)	Height (25282103)	0.0002017	0.015311661
**rs283553**	intergenic	TFAP2B, PKHD1	chr6	**LD**	Confirmed, novel	1.69E-05	0.015442775
**rs2677031**	intergenic	CALCR, MIR4652	chr7	Novel	Confirmed, novel	1.63E-05	0.01561346
**rs12104582**	intergenic	LOC400940,	chr2	**LD**	Confirmed, hip circumference	0.0001975	0.01570125

		LINC01247			(25673412)		
**rs2770102**	ncRNA_intronic	CASC15	chr6	Novel	Confirmed	1.66E-05	0.01586085
**rs3776569**	intronic	SLC1A3	chr5	Novel	Novel	4.80E-05	0.015938517
**rs6060001**	intronic	TP53INP2	chr20	Height (25282103)	Height (25282103)	0.0003549	0.01604655
**rs3818717**	exonic	RAI1	chr17	Novel	Novel	0.0003603	0.016049727
**rs12129408**	intergenic	ADGRL2, LINC01361	chr1	Novel	Novel, novel	1.97E-05	0.016094004
**rs889203**	intronic	GPR139	chr16	**LD**	Confirmed	3.04E-05	0.016683789
**rs10775267**	intergenic	GPR139, GP2	chr16	Novel	Confirmed, confirmed	0.0002636	0.01690335
**rs11674696**	intergenic	MIR1302-4, CREB1	chr2	Novel	Novel, confirmed	2.76E-05	0.017081996
**rs10271989**	ncRNA_intronic	LOC101928782	chr7	Novel	Novel	0.0001902	0.017264308
**rs1320252**	intergenic	MAP2K3, KCNJ12	chr17	**LD**	Confirmed, confirmed	3.55E-05	0.017606529
**rs2903940**	intronic	EYA2	chr20	Novel	Confirmed	0.0002556	0.017729345
**rs9322955**	intergenic	NFKBIA, INSM2	chr14	Novel	Confirmed, novel	2.74E-05	0.019324319
**rs10514944**	intronic	SKAP1	chr17	Novel	Confirmed	0.0002798	0.019332431
**rs231976**	intergenic	IL27, NUPR1	chr16	Novel	Novel, novel	2.14E-05	0.019340286
**rs2457831**	intergenic	INADL, L1TD1	chr1	Novel	Confirmed, novel	2.27E-05	0.019389345
**rs6904716**	intronic	LEMD2	chr6	Novel	Confirmed	0.0001121	0.019463133
**rs3759579**	upstream	MARK3	chr14	Novel	Novel	2.18E-05	0.019637877
**rs836525**	intronic	DAGLB	chr7	Novel	Novel	5.83E-05	0.019785293
**rs941737**	intergenic	STXBP6, NOVA1	chr14	Novel	Confirmed, confirmed	8.98E-05	0.020287948

**rs12406019**	intergenic	GIPC2, MGC27382	chr1	Hip circumference in females (25673412)	Height (25282103), novel	2.61E-05	0.021112019
**rs6766759**	intronic	FAM19A1	chr3	Novel	Novel	5.55E-05	0.021609989
**rs2524099**	downstream	HLA-C	chr6	Novel	Novel	2.63E-05	0.022061256
**rs10114139**	intergenic	STRBP, CRB2	chr9	Novel	Novel, confirmed	0.0003249	0.02239245
**rs6867577**	intronic	FBXL17	chr5	Novel	Confirmed	4.18E-05	0.022967669
**rs756717**	intronic	ZFHX3	chr16	Novel	Confirmed	7.85E-05	0.022986011
**rs11037685**	intergenic	HSD17B12, ALKBH3	chr11	Novel	Confirmed, novel	8.55E-05	0.02308269
**rs11100760**	intronic	INPP4B	chr4	Novel	Novel	0.0001477	0.023176989
**rs9450537**	intergenic	SNHG5, HTR1E	chr6	Novel	Novel, novel	0.0001392	0.023706533
**rs11075994**	intronic	FTO	chr16	Novel	Confirmed	0.0001272	0.0240408
**rs11755266**	intergenic	TCP11, SCUBE3	chr6	Height (23754948)	Height (18391951)	9.96E-05	0.024225664
**rs17667540**	ncRNA_intronic	SCHLAP1	chr2	Novel	Confirmed	0.0001953	0.024264217
**rs2273684**	intronic	GSS	chr20	Height (25282103)	Height (25282103)	0.0003858	0.024621055
**rs2280406**	upstream	MST1R	chr3	Novel	Novel	5.63E-05	0.02479517
**rs12436181**	intronic	TRAF3	chr14	Novel	Confirmed	0.0001721	0.024848825
**rs12912380**	intronic	SEMA6D	chr15	Novel	Confirmed	5.63E-05	0.0251098
**rs12245680**	intronic	TCF7L2	chr10	T2D (24509480)	Confirmed	0.01444	0.02527
**rs1015055**	intronic	NTM	chr11	Novel	Confirmed	0.0003049	0.025481855
**rs10838708**	intronic	PSMC3	chr11	Height (25282103)	Height (25282103)	3.63E-05	0.025743288
**rs1604038**	intergenic	EIF5A2, SLC2A2	chr3	Fasting glucose (22885924)	Novel, confirmed	0.001247	0.026394833

**rs9512670**	intergenic	RASL11A, GTF3A	chr13	Novel	Novel, confirmed	0.0001385	0.026899036
**rs7612543**	intronic	ZBTB38	chr3	Height (25282103)	Confirmed	0.0001385	0.027807901
**rs500422**	UTR5	USP37	chr2	Height (25282103)	Confirmed	3.49E-05	0.028400844
**rs12923427**	intergenic	XYLT1, NPIPA8	chr16	Novel	Novel, novel	0.0006042	0.028822578
**rs716591**	intergenic	MCTP2, LOC440311	chr15	Novel	Confirmed, novel	0.0003772	0.028859498
**rs1593374**	ncRNA_intronic	LOC102724623	chr8	Novel	Confirmed	0.000196	0.029176438
**rs1500486**	intergenic	GNPDA2, GABRG1	chr4	Novel	Confirmed, novel	6.33E-05	0.029356162
**rs17231277**	intergenic	LOC101928435, PSMG1	chr21	Novel	Novel, novel	3.68E-05	0.029578488
**rs2886070**	intergenic	ARHGEF2, SSR2	chr1	Novel	Novel, novel	0.0002133	0.030396672
**rs10470517**	intergenic	DNAJC19, SOX2-OT	chr3	Novel	Novel, novel	9.86E-05	0.030676193
**rs1434285**	intergenic	AMIGO1, GPR61	chr1	Novel	Novel, confirmed	0.0001318	0.031080446
**rs7689420**	intronic	HHIP	chr4	Height (25282103)	Confirmed	0.000124	0.031907087
**rs879070**	intronic	DENND1A	chr9	Novel	Confirmed	0.0009762	0.031957705
**rs2488071**	intergenic	HHEX, EXOC6	chr10	Novel	Confirmed, confirmed	0.006642	0.032103
**rs16850638**	intergenic	MIR548A3, ALCAM	chr3	Novel	Novel, confirmed	0.00012	0.032544255
**rs6747219**	ncRNA_intronic	LINC01122	chr2	Novel	Confirmed	0.0002862	0.032587773

**rs2306369**	exonic	KIAA1109	chr4	Novel	Novel	0.0002967	0.033628952
**rs704084**	intergenic	DUSP6, POC1B	chr12	Novel	Novel, novel	5.39E-05	0.034126828
**rs11979110**	intergenic	KLF14, MIR29A	chr7	HDL/HDL cholesterol (24097068)	Confirmed, novel	0.00288	0.03456
**rs2862299**	intronic	SEC16B	chr1	Novel	Confirmed, confirmed	0.0001289	0.03521574
**rs621042**	intronic	PIK3C2G	chr12	Novel	Confirmed	5.03E-05	0.035303938
**rs2212450**	intergenic	LOC387810, LOC101928847	chr11	Novel	Novel, novel	5.03E-05	0.035350484
**rs10853100**	intergenic	LOC101927166, HOXB1	chr17	Novel	Novel, novel	5.63E-05	0.035800418
**rs6474952**	intronic	CCDC171	chr9	**LD**	Confirmed	0.0001975	0.035949202
**rs12050677**	intronic	OTUD7A	chr15	Novel	Novel	0.0004653	0.0359691
**rs3814614**	intergenic	GRID1, WAPL	chr10	Novel	Confirmed, novel	0.001154	0.0360625
**rs732310**	intronic	REXO1	chr19	Novel	Novel	0.0001545	0.036143822
**rs4329699**	intronic	NTM	chr11	Novel	Confirmed	0.0004894	0.036563986
**rs2100814**	intergenic	ELP3, PNOC	chr8	Novel	Confirmed, novel	0.001864	0.036993231
**rs10898868**	intronic	ARAP1	chr11	log(Proinsulin) (21873549)	Confirmed	0.002589	0.0370227
**rs214246**	intronic	AXIN1	chr16	Novel	Confirmed	0.0001545	0.037419608
**rs6500208**	intergenic	MIR5095, CBLN1	chr16	Novel	Novel, confirmed	5.56E-05	0.037479969
**rs834027**	intronic	UBAP2	chr9	Novel	Novel	0.001299	0.037598833
**rs2505276**	intergenic	TFAP2B, PKHD1	chr6	**LD**	Confirmed, novel	5.16E-05	0.037811686
**rs3803522**	ncRNA_exonic	LOC101929076	chr15	Novel	Novel	0.0001039	0.039104627

**rs1078101**	intergenic	HAAO, LOC102723854	chr2	Novel	Novel, novel	0.0001768	0.0392548
**rs6021796**	intergenic	ZFP64, LINC01524	chr20	Novel	Confirmed, novel	0.0001768	0.03926728
**rs12066815**	intronic	NTNG1	chr1	Novel	Novel	9.19E-05	0.03936521
**rs4414033**	intergenic	C1orf61, MEF2D	chr1	Novel	Novel, novel	7.78E-05	0.039446552
**rs4978760**	intronic	IKBKAP	chr9	Novel	Novel	0.0005474	0.039728251
**rs13337591**	intronic	FTO	chr16	Novel	Confirmed	6.53E-05	0.039910651
**rs2550739**	intergenic	MIR4720, CMIP	chr16	Novel	Novel, confirmed	0.0001472	0.039915508
**rs4809440**	intergenic	SLC17A9, BHLHE23	chr20	Novel	Novel, novel	0.0005606	0.040829051
**rs8001047**	intergenic	PDS5B, LINC00423	chr13	Novel	Confirmed, novel	0.001864	0.040883733
**rs11815022**	intronic	PAX2	chr10	Novel	Confirmed	6.33E-05	0.040918054
**rs7020269**	intergenic	C9orf72, LINGO2	chr9	Novel	Novel, confirmed	0.001427	0.041065889
**rs1037954**	ncRNA_intronic	LOC101927314, MIR548H3	chr6	Novel	Confirmed, novel	6.33E-05	0.041476895
**rs7801936**	intronic	FKBP6	chr7	Novel	Novel	0.0007047	0.041705427
**rs1993414**	intergenic	TMEM26-AS1, C10orf107	chr10	Novel	Novel, novel	0.0001133	0.041955785
**rs1320366**	intergenic	FAM150B, TMEM18	chr2	**LD**	Novel, confirmed	6.87E-05	0.042030092

**rs7821182**	intergenic	LINC01419, RALYL	chr8	**LD**	Novel, confirmed	6.33E-05	0.042750496
**rs8101491**	intronic	SAE1	chr19	Novel	Novel	8.74E-05	0.043578635
**rs1008007**	intronic	LTBP2	chr14	Novel	Novel	7.12E-05	0.044755282
**rs3750450**	exonic	EPB41L4B	chr9	Novel	Confirmed	0.0007529	0.045174
**rs305002**	intergenic	LINC00593, TLE3	chr15	Novel	Novel, novel	0.0006339	0.045332654
**rs7972585**	intergenic	MVK, FAM222A	chr12	Novel	Novel, novel	9.42E-05	0.045396751
**rs3784635**	exonic	VPS13C	chr15	Novel	Confirmed	0.001758	0.0455908
**rs2018218**	intronic	NCR3LG1	chr11	Novel	Novel	0.0006148	0.045596263
**rs2710508**	intronic	EPHA4	chr2	Novel	Confirmed	6.85E-05	0.045740872
**rs10228276**	downstream	HOTTIP	chr7	Novel	Novel	0.0001161	0.046225662
**rs11211176**	intergenic	IPP, MAST2	chr1	Height (25282103)	Novel, confirmed	0.0004653	0.046248371
**rs534523**	intergenic	MIR124-1, MSRA	chr8	Novel	Novel, adiposity (19557161)	0.001566	0.0466668
**rs605765**	intergenic	KCNA4, FSHB	chr11	Novel	Novel, confirmed	0.0002843	0.046693314
**rs2485534**	intronic	GRIN3A	chr9	Novel	Novel	0.001696	0.0468096
**rs4678856**	intergenic	ARPP21, STAC	chr3	Novel	Novel, confirmed	8.00E-05	0.047525111
**rs4474658**	intergenic	C2CD4A, C2CD4B	chr15	log(Proinsulin) (21873549)	Height (20881960), confirmed	0.009024	0.048987429
**rs1438995**	intergenic	LINC00936, LINC00615	chr12	Novel	Novel, novel	9.72E-05	0.048996122
**rs3911565**	intronic	FAT3	chr11	Novel	Glucose homeostasis traits (25524916)	0.0006653	0.049335883

**rs4937729**	intronic	OPCML	chr11	Novel	Novel	0.001046	0.049390813
**rs2275003**	intronic	DCAF12	chr9	Novel	Novel	0.0004117	0.049943469

Notes:

SNP type means whether SNPs identified in our study compared to the original BMI GWAS and previous studies are Novel or Confirmed or associated with BMI-related traits (trait (PMID)) or in high LD with BMI-associated loci.

Gene type means whether genes identified in our study compared to the original BMI GWAS and previous studies are Novel or Confirmed.

P.valueA is the p value of BMI, A is BMI.

cFDR.AcB is the cFDR value of BMI conditioned on T2D, B is T2D.

Table S3 SNPs in high LD (R^2 > 0.6) with BMI-associated loci

SNP	R2	Proxy SNP	PMID	Trait
**rs2235569**	0.962	rs2814992	GIANT (25673413)	BMI
**rs17243334**	0.675	rs997295	GIANT (25673413)	BMI
**rs7503807**	0.967	rs12940622	GIANT (25673413)	BMI
**rs4949662**	0.933	rs17381664	GIANT (25673413)	BMI
**rs11632574**	0.677	rs8032675	GIANT (25673413)	BMI
**rs10840103**	0.848	rs4929949	Wheeler E (23563609)	Extreme obesity with early age of onset
**rs1861569**	0.933	rs2908884	GIANT (25673413)	BMI
**rs1549979**	0.665	rs12637027	GIANT (25673413)	BMI
**rs9784046**	0.633	rs12714412	GIANT (25673413)	BMI
**rs9381901**	1	rs2744489	GIANT (25673413)	BMI
**rs9408902**	1	rs1928295	GIANT (25673413)	BMI
**rs12127789**	0.851	rs12134600	GIANT (25673413)	BMI
**rs7232886**	1	rs8085349	GIANT (25673413)	BMI
**rs4771946**	0.818	rs9634490	GIANT (23563607)	Obesity
**rs9659092**	0.836	rs11583200	GIANT (25673413)	BMI
**rs7228347**	0.789	rs17066856	GIANT (25673413)	BMI
**rs17326595**	0.647	rs448376	GIANT (23563607)	Overweight
**rs3814878**	0.65	rs12325539	GIANT (25282103)	height
**rs283553**	0.658	rs4711966	GIANT (25673413)	BMI
**rs12104582**	0.964	rs10929925	GIANT (25673412)	Hip circumference

**rs889203**	0.774	rs11074422	GIANT (25673413)	BMI
**rs1320252**	0.866	rs1914888	GIANT (25282103)	Height
**rs6474952**	0.755	rs4740619	GIANT (25673413)	BMI
**rs2505276**	0.655	rs12529657	GIANT (25673413)	BMI
**rs1320366**	0.737	rs10209658	GIANT (25673413)	BMI
**rs7821182**	0.726	rs2033732	GIANT (25673413)	BMI

Table S4 Conditional FDR value for T2D given the BMI (cFDR < 0.05).

RSID	ROLE	GENE	CHR	SNP type	Gene type	P.valueB	cFDR.BcA
**rs10787472**	intronic	TCF7L2	chr10	Confirmed	Confirmed	1.30E-36	6.63E-35
**rs4481184**	intronic	IGF2BP2	chr3	Confirmed	Confirmed	4.50E-22	6.55E-20
**rs6906327**	intronic	CDKAL1	chr6	Confirmed	Confirmed	1.10E-23	1.82E-19
**rs9930506**	intronic	FTO	chr16	BMI (25673413)	Confirmed	1.90E-10	1.90E-10
**rs849135**	intronic	JAZF1	chr7	T2D (22885922)	Confirmed	1.70E-09	7.08E-08
**rs2881654**	intronic	PPARG	chr3	Confirmed	Confirmed	3.40E-09	7.82E-08
**rs12245680**	intronic	TCF7L2	chr10	Confirmed	Confirmed	1.10E-09	6.70E-07
**rs163177**	intronic	KCNQ1	chr11	Confirmed	Confirmed	2.00E-10	2.10E-06
**rs4430796**	intronic	HNF1B	chr17	T2D (26551672)	Confirmed	8.90E-10	9.19E-06
**rs6103716**	intronic	HNF4A	chr20	Confirmed	Confirmed	6.90E-09	1.27E-05
**rs10811658**	intergenic	CDKN2B-AS1, DMRTA1	chr9	Confirmed	Confirmed	5.00E-08	9.08E-05
**rs12624485**	intergenic	FITM2, R3HDML	chr20	Confirmed	Confirmed	2.00E-08	0.000131
**rs4812831**	ncRNA_intronic	HNF4A-AS1	chr20	Novel	Novel	1.20E-07	0.000209
**rs7119**	UTR3	HMG20A	chr15	Novel	Confirmed	6.50E-08	0.000264
**rs3843467**	intronic	C5orf67	chr5	Triglycerides (20686565)	Novel	2.40E-07	0.000293
**rs7079711**	intronic	TCF7L2	chr10	Novel	Confirmed	2.40E-07	0.000339
**rs4753073**	intergenic	MTNR1B, SLC36A4	chr11	Fasting glucose (20081858)	Confirmed, novel	5.70E-07	0.000373
**rs17013383**	ncRNA_intronic	MIR548AC	chr3	T2D (22158537)	Confirmed	5.50E-07	0.000549

**rs2283228**	intronic	KCNQ1	chr11	**LD**	Confirmed	7.20E-08	0.000628
**rs2488071**	intergenic	HHEX, EXOC6	chr10	Novel	Confirmed	4.70E-06	0.001119
**rs7141420**	intronic	NRXN3	chr14	BMI (25673413)	BMI (25673413)	0.00025	0.001125
**rs6885904**	ncRNA_intronic	ZBED3-AS1	chr5	Novel	Confirmed	1.50E-06	0.00173
**rs4686698**	intronic	IGF2BP2	chr3	Novel	Confirmed	1.70E-06	0.001765
**rs231354**	ncRNA_exonic	KCNQ1OT1	chr11	Novel	Confirmed	4.60E-07	0.001934
**rs11514706**	intergenic	DGKB, AGMO	chr7	Fasting glucose (20081858)	Confirmed, confirmed	2.20E-06	0.002021
**rs3130931**	UTR5	POU5F1	chr6	Height (25282103)	Confirmed	5.10E-06	0.002127
**rs10885414**	intronic	TCF7L2	chr10	Novel	Confirmed	1.00E-05	0.003658
**rs2291720**	intergenic	ZNF385D-AS2, UBE2E2	chr3	T2D (22158537)	Confirmed, confirmed	4.60E-06	0.003868
**rs6991067**	intronic	INTS8	chr8	**LD**	Confirmed	3.00E-06	0.005432
**rs12217971**	intergenic	CDC123, CAMK1D	chr10	Novel	Confirmed	8.70E-06	0.005586
**rs6795735**	ncRNA_intronic	ADAMTS9-AS2	chr3	T2D (22885922)	Confirmed	2.00E-04	0.00604
**rs17150816**	intergenic	MIR124-1, MSRA	chr8	Novel	Novel, novel	5.50E-06	0.006156
**rs12895330**	intergenic	AKAP6, NPAS3	chr14	Novel	Novel, novel	0.00021	0.007245
**rs3892710**	intergenic	HLA-DQB1, HLA-DQA2	chr6	T1D (17632545)	Confirmed, confirmed	2.10E-05	0.007928
**rs234857**	intronic	KCNQ1	chr11	Novel	Confirmed	8.50E-06	0.008189
**rs17791633**	intergenic	LOC101927450, TLE4	chr9	**LD**	Confirmed, confirmed	1.90E-06	0.008887
**rs7965349**	intronic	OASL	chr12	**LD**	Confirmed	2.80E-06	0.008893
**rs1019029**	intergenic	ARL4A, ETV1	chr7	Novel	Novel, novel	1.10E-05	0.009012

**rs2327778**	intergenic	SLC35D3, NHEG1	chr6	Novel	Confirmed, novel	5.30E-06	0.009304
**rs2789686**	UTR3	ANXA11	chr10	Novel	Novel	1.30E-05	0.01137
**rs7812465**	intronic	NDUFAF6	chr8	**LD**	Confirmed	2.40E-06	0.011919
**rs11659412**	intronic	LAMA1	chr18	**LD**	Confirmed	8.60E-06	0.013306
**rs17013152**	ncRNA_intronic	MIR548AC	chr3	**LD**	Novel	2.70E-05	0.013819
**rs11979110**	intergenic	KLF14, MIR29A	chr7	HDL (20686565)	Confirmed, novel	9.70E-05	0.014097
**rs2540074**	intronic	STRBP	chr9	Novel	Novel	6.50E-05	0.014357
**rs7026633**	intergenic	LOC101927450, TLE4	chr9	Novel	Confirmed, confirmed	1.00E-05	0.017157
**rs290483**	intronic	TCF7L2	chr10	Novel	Confirmed	1.70E-05	0.017162
**rs530703**	intergenic	NPBWR2, MYT1	chr20	Novel	Novel, novel	4.60E-05	0.017204
**rs7005140**	intergenic	SLC30A8, MED30	chr8	Fasting glucose (22885924)	Confirmed, novel	1.90E-05	0.0176
**rs2334255**	UTR3	GIPR	chr19	Novel	BMI (20935630)	0.00034	0.017642
**rs4712540**	intronic	CDKAL1	chr6	T2D (26551672)	Confirmed	8.00E-06	0.017913
**rs2466292**	downstream	SLC30A8	chr8	T2D (26551672)	Confirmed	1.70E-05	0.018263
**rs1783598**	intronic	FCHSD2	chr11	log(Proinsulin) (21873549)	Novel	0.00052	0.019356
**rs2190324**	intergenic	ARL4A, ETV1	chr7	Novel	Novel, novel	9.20E-06	0.021039
**rs11548393**	UTR3	SPRED2	chr2	Novel	Novel	1.10E-05	0.023013
**rs17584208**	intergenic	PSRC1, MYBPHL	chr1	LDL (20686565)	LDL GLGC(20686565), novel	0.0016	0.02976
**rs9349534**	intergenic	DEFB112, TFAP2D	chr6	Novel	Novel, novel	4.50E-05	0.03216
**rs7748736**	intronic	SSR1	chr6	Novel	Confirmed	3.20E-05	0.035146

**rs1473**	intronic	PUM1	chr1	Novel	Novel	0.00092	0.03542
**rs10898868**	intronic	ARAP1	chr11	log(Proinsulin) (21873549)	Confirmed	0.00044	0.03608
**rs11639314**	intronic	PEAK1	chr15	**LD**	Novel	3.90E-05	0.036597
**rs1996023**	intergenic	GNPDA2, GABRG1	chr4	BMI (25673413)	BMI (25673413), novel	0.025	0.0375
**rs10743609**	intergenic	KLHL42, PTHLH	chr12	Novel	Novel, novel	1.60E-05	0.038647
**rs3898882**	intergenic	BCL2L11, MIR4435-1	chr2	**LD**	Novel, novel	0.00011	0.038885
**rs9268402**	intergenic	C6orf10, HCG23	chr6	Novel	Novel, novel	0.00013	0.04056
**rs7186510**	intronic	CMIP	chr16	Novel	Novel	3.00E-04	0.044373
**rs825461**	intronic	ZNF664, FAM101A	chr12	HDL (24097068)	Waist-to-hip ratio adjusted for BMI (25673412)	0.0013	0.04472
**rs7586601**	intergenic	GTF3C2, EIF2B4	chr2	Triglycerides (20686565)	Novel, novel	0.00024	0.0449
**rs192710**	intergenic	TSPAN15, NEUROG3	chr10	Novel	Novel, novel	5.80E-05	0.044956
**rs16945088**	intronic	FTO	chr16	BMI (25673413)	Confirmed	0.0072	0.045
**rs9540493**	intergenic	LOC102723968, LINC01052	chr13	BMI (25673413)	Novel, novel	0.0057	0.0456
**rs11247980**	intergenic	CTBP1-AS2, MAEA	chr4	Novel	Confirmed, confirmed	6.60E-05	0.047601
**rs4238585**	intergenic	GPR139, GP2	chr16	BMI (25673413)	Novel, BMI (22344219)	0.0069	0.0483
**rs12454712**	intronic	BCL2	chr18	T2D (22325160)	Confirmed	0.0034	0.048571

**rs1147322**	UTR5	ZBTB6	chr9	Novel	Novel	0.00039	0.048927

Notes:

SNP type means whether SNPs identified in our study compared to the original T2D GWAS and previous studies are Novel or Confirmed or associated with T2D-related traits (trait (PMID)) or in high LD with T2D-associated loci.

Gene type means whether genes identified in our study compared to the original T2D GWAS and previous studies are Novel or Confirmed.

P.valueB is the p value of T2D, B is T2D.

cFDR.BcA is the cFDR value of T2D conditioned on BMI, A is BMI.

Table S5 SNPs in high LD (R^2 > 0.6) with T2D-associated loci

SNP	R2	Proxy SNP	PMID	Trait
**rs2283228**	1	rs2237892	DIAGRAM (24509480)	T2D
**rs6991067**	0.621	rs13257021	DIAGRAM (24509480)	T2D
**rs17791633**	0.785	rs13292136	Voight BF (20581827)	T2D
**rs7965349**	0.817	rs7957197	Voight BF (20581827)	T2D
**rs7812465**	0.688	rs10099941	DIAGRAM (24509480)	T2D
**rs11659412**	0.851	rs8090011	Perry JR (22693455)	T2D
**rs17013152**	1	rs17012998	Cho YS (22158537)	T2D
**rs11639314**	0.606	rs12911371	DIAGRAM (24509480)	T2D
**rs3898882**	0.813	rs7565457	GIANT (25673413)	Height

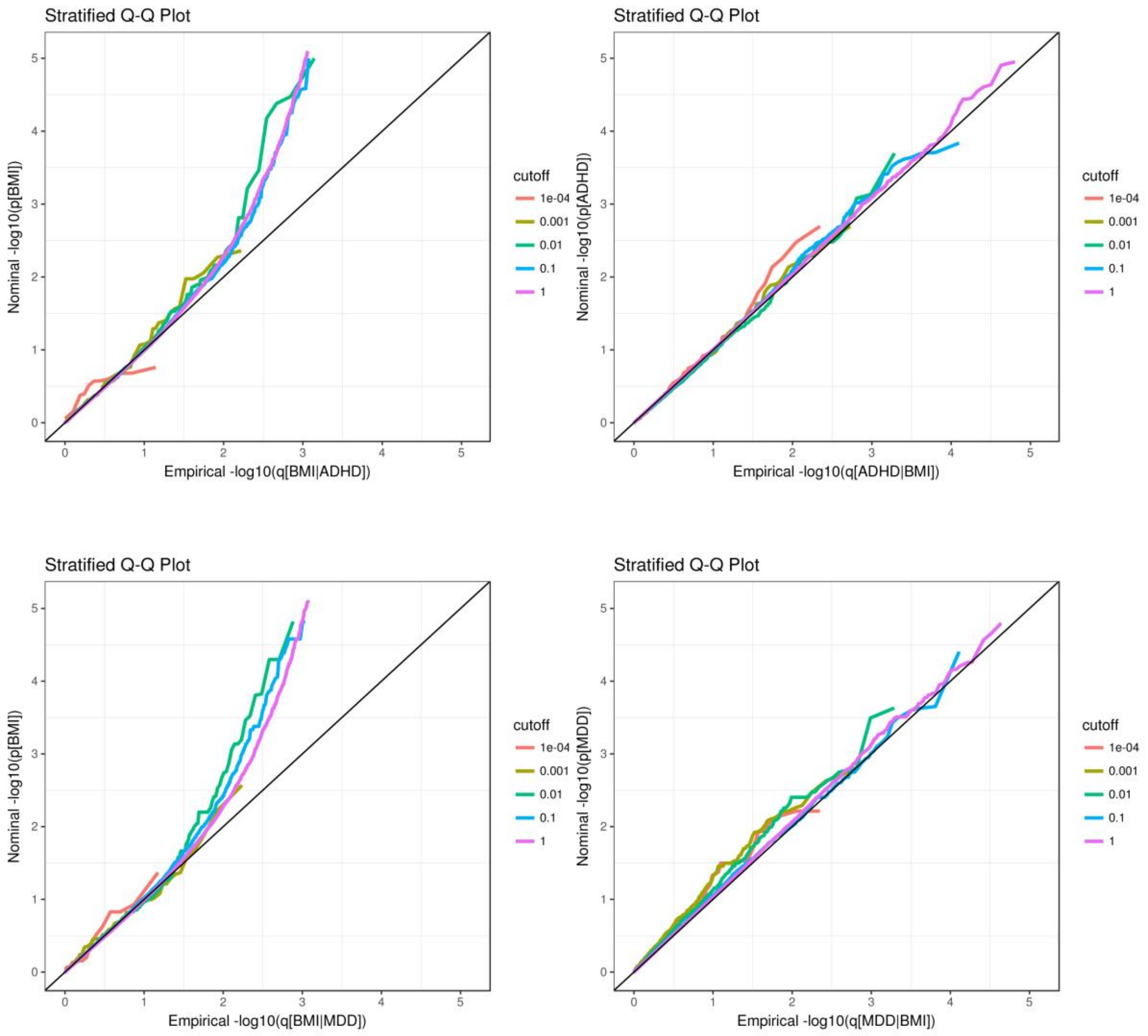

Figure S1. Stratified QQ plots. Upper Panel: Stratified QQ plots of nominal versus empirical $-\log_{10}$ p-values in (left) BMI as a function of significance of the association with ADHD, and in (right) ADHD as a function of significance of the association with BMI. Lower Panel: Stratified QQ plots of nominal versus empirical $-\log_{10}$ p-values in (left) BMI as a function of significance of the association with MDD, and in (right) MDD as a function of significance of the association with BMI.

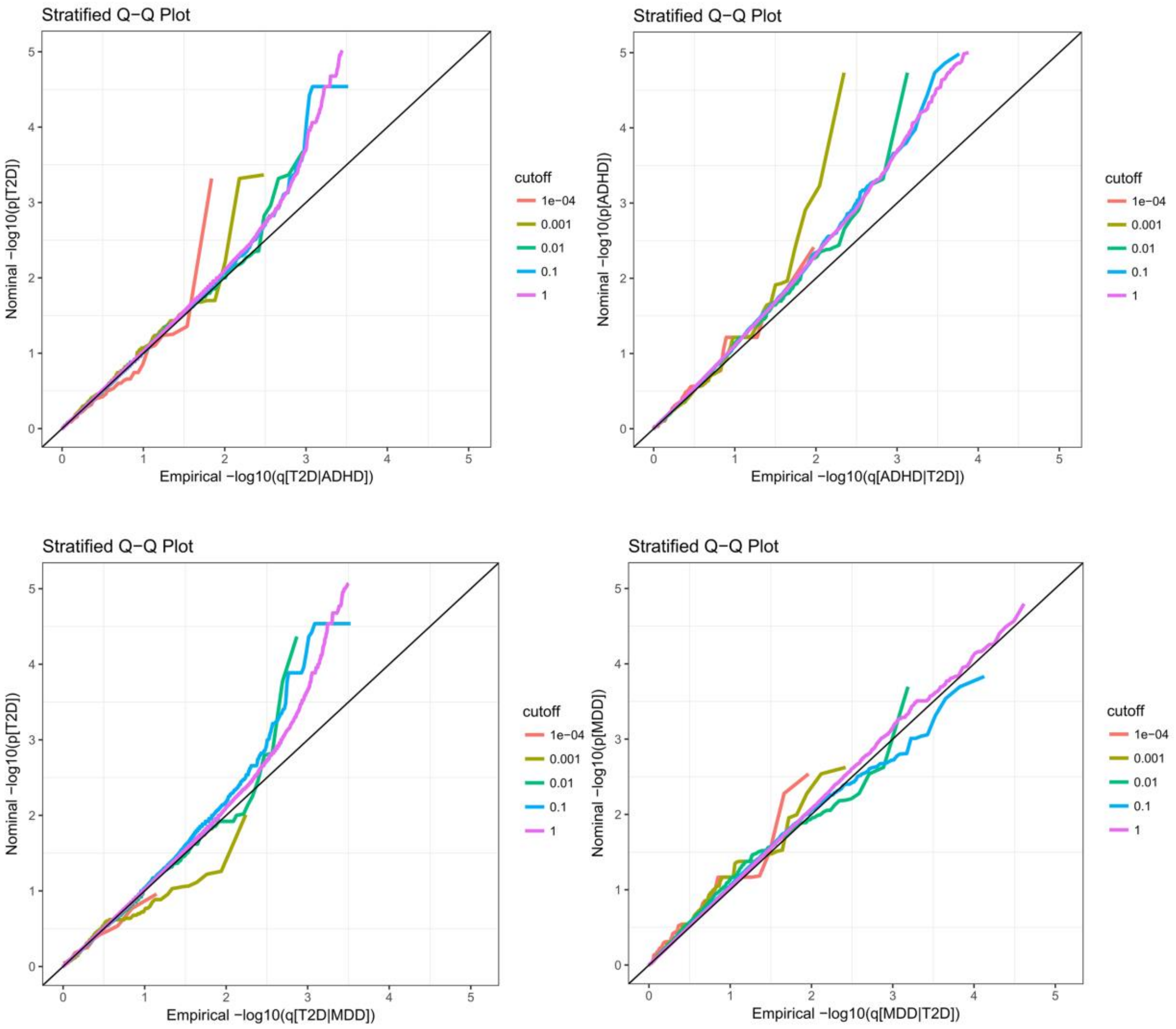

Figure S2. Stratified QQ plots. Upper Panel: Stratified QQ plots of nominal versus empirical $-\log_{10}$ p-values in (left) T2D as a function of significance of the association with ADHD, and in (right) ADHD as a function of significance of the association with T2D. Lower Panel: Stratified QQ plots of nominal versus empirical $-\log_{10}$ p-values in (left) T2D as a function of significance of the association with MDD, and in (right) MDD as a function of significance of the association with T2D.

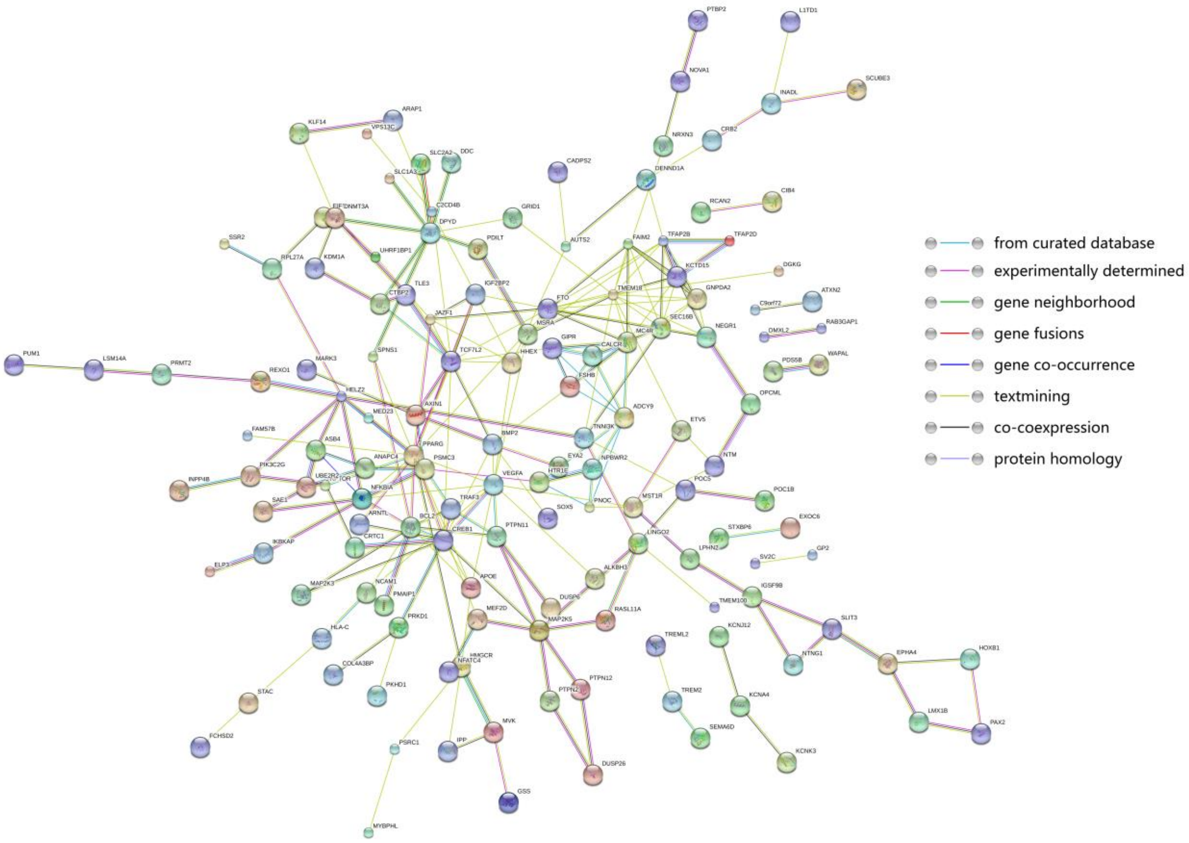

Figure S3. Protein-protein interactions between BMI-associated genes.

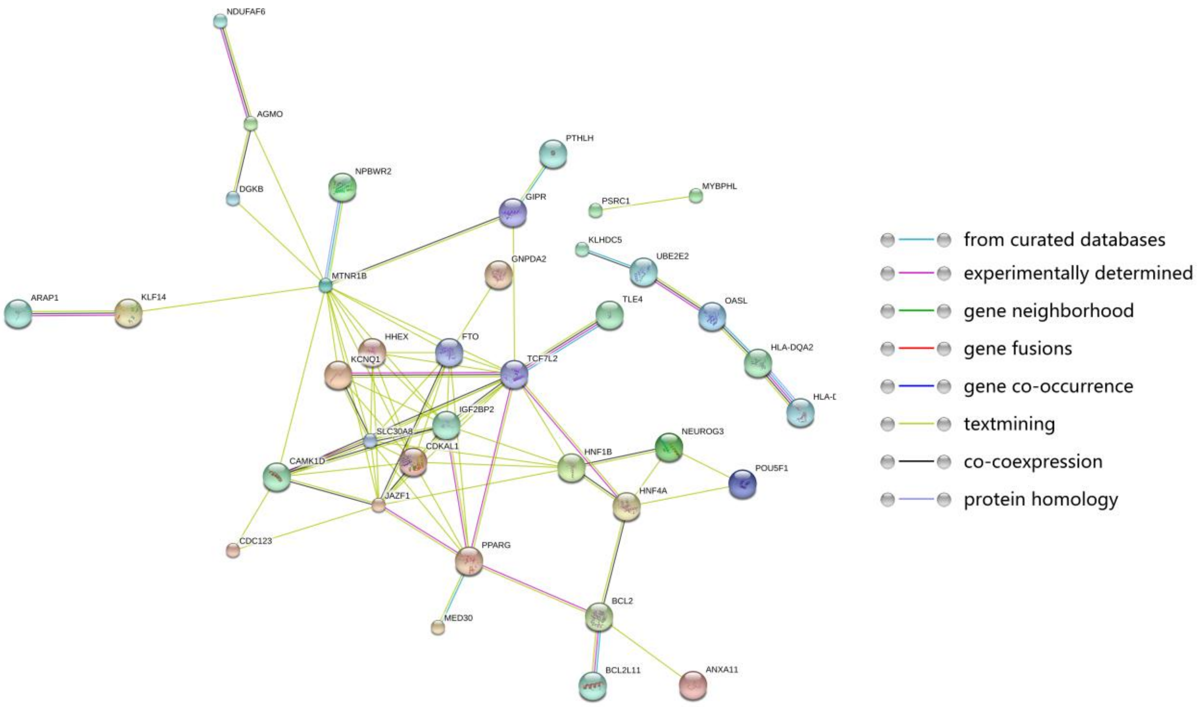

Figure S4. Protein-protein interactions between T2D-associated genes.
